# Mutagenesis and structural studies reveal the basis for the specific binding of SARS-CoV-2 SL3 RNA element with human TIA1 protein

**DOI:** 10.1038/s41467-023-39410-8

**Published:** 2023-06-22

**Authors:** Dong Zhang, Lulu Qiao, Xiaobo Lei, Xiaojing Dong, Yunguang Tong, Jianwei Wang, Zhiye Wang, Ruhong Zhou

**Affiliations:** 1 Institute of Quantitative Biology, College of Life Sciences, Zhejiang University, Hangzhou, Zhejiang 310058 China; 2 State Key Laboratory of Plant Physiology and Biochemistry, College of Life Sciences, Zhejiang University, Hangzhou, Zhejiang 310058 China; 3 NHC Key Laboratory of Systems Biology of Pathogens and Christophe Mérieux Laboratory, Institute of Pathogen Biology, Chinese Academy of Medical Sciences & Peking Union Medical College, Beijing, 100730 China; 4 College of Life Sciences, China Jiliang University, Hangzhou, Zhejiang 310018 China; 5 Department of Pharmacy, China Jiliang University, Hangzhou, Zhejiang 310018 China; 6 The First Affiliated Hospital, College of Medicine, Zhejiang University, Hangzhou, Zhejiang 310058 China

**Keywords:** Computational biophysics, Biological physics

## Abstract

Viral RNA-host protein interactions are indispensable during RNA virus transcription and replication, but their detailed structural and dynamical features remain largely elusive. Here, we characterize the binding interface for the SARS-CoV-2 stem-loop 3 (SL3) cis-acting element to human TIA1 protein with a combined theoretical and experimental approaches. The highly structured SARS-CoV-2 SL3 has a high binding affinity to TIA1 protein, in which the aromatic stacking, hydrogen bonds, and hydrophobic interactions collectively direct this specific binding. Further mutagenesis studies validate our proposed 3D binding model and reveal two SL3 variants have enhanced binding affinities to TIA1. And disruptions of the identified RNA-protein interactions with designed antisense oligonucleotides dramatically reduce SARS-CoV-2 infection in cells. Finally, TIA1 protein could interact with conserved SL3 RNA elements within other betacoronavirus lineages. These findings open an avenue to explore the viral RNA-host protein interactions and provide a pioneering structural basis for RNA-targeting antiviral drug design.

## Abstract (web summary)

Here the authors reveal the binding interface for the SARS-CoV-2 SL3 RNA element to human TIA1 protein and find that disruptions of the identified viral RNA-host protein interactions with designed ASOs reduce SARS-CoV-2 infection in cells.

## Introduction

There are hundreds of different RNA viruses that trigger infectious diseases in the world, such as the ongoing COVID-19 pandemic caused by the severe acute respiratory syndrome coronavirus 2 (SARS-CoV-2)^1,2^. Due to the RNA nature of their genomes, it is anticipated that RNA viruses would co-opt host RNA-binding proteins (RBPs) to perform their particular functions during transcription and replication^3–5^. For instance, several host RBPs have been identified that affect the recruitment of plus viral strands for replication, play important roles during the switch from translation to replication, control viral RNA synthesis, affect the stability of viral RNAs, and participate in multiple events during infection^3^. Therefore, a deeper understanding of the interactions between viral RNAs and host RBPs will provide new insights into the virus life cycle and help the development of novel antiviral strategies.

During the last few years, in order to understand and combat SARS-CoV-2, various ‘omics’ technologies have been applied to discover the proviral host factors that are required for the completion of the SARS-CoV-2 life cycle^6–9^. For example, Flynn et al.^7^ identified 309 host proteins that bind the SARS-CoV-2 RNA during active infection by mass spectrometry (ChIRP-MS) and provided a comprehensive catalog of functional SARS-CoV-2 RNA-host protein interactions. Schmidt et al.^9^ identified up to 104 human proteins that directly and specifically bind to SARS-CoV-2 RNAs in infected human cells by using RNA antisense purification coupling with mass spectrometry, and linked protein-RNA interactome to cellular pathways relevant to SARS-CoV-2 infections. Kamel et al.^8^ identified comprehensively the RBPs involved in SARS-CoV-2 infection through a multi-omic approach and revealed that SARS-CoV-2 infection profoundly remodels the cellular RNA-bound proteome. Overall, the molecular functions of the related genes encoding proteins identified as binding partners for viral RNA include mRNA processing stability and localization, P-body/stress granule assembly, translation, and many more^6^. Some representative host proteins that have been individually validated as proviral factors include dead-box helicase 5 (DDX5), T-cell intracellular antigen-1 (TIA1), and insulin like growth factor 2 mRNA binding protein 1^6^. Nonetheless, which segment of the SARS-CoV-2 RNA genome serves as the binding site for a particular host protein remains largely elusive.

Generally, the genome of positive-sense RNA viruses, including SARS-CoV-2, encode the information essential for their life cycle in two aspects. Apart from the common coding regions for the translation of proteins that hijack the host cell machinery and assemble new viral particles, the single-stranded RNA genome could fold back onto itself to form the so-called cis-acting elements in the 5′- and 3′-untranslated regions (UTR)^5,10–12^. Previous studies^5,11,12^ have demonstrated that those cis-acting elements are essential for viral transcription and replication through complicated RNA–RNA and RNA–protein interactions. Particularly, the 5′-UTR of the SARS-CoV-2 RNA genome is highly structured and harbors several stem-loop (SL) elements (such as SL1, SL2, SL3, etc.) conserved among the betacoronavirus lineage, which has been validated by chemical probing, NMR chemical shift experiments, and computational predictions^13–19^. Moreover, the secondary structures of the isolated SL cis-acting elements are in good agreement with the full-length construct^16^, implying their ability to fold independently. Furthermore, compared to the high mutation rates in the coding regions, those SL elements are thought to be highly sequence and structure conserved in different virus variants^11,12^. Therefore, those properties make the SL cis-acting elements attractive targets for development of novel antivirals, such as the RNA-targeting antivirals, against the rapidly evolved RNA viruses and the possible future coronavirus outbreaks.

**Fig. 1:**
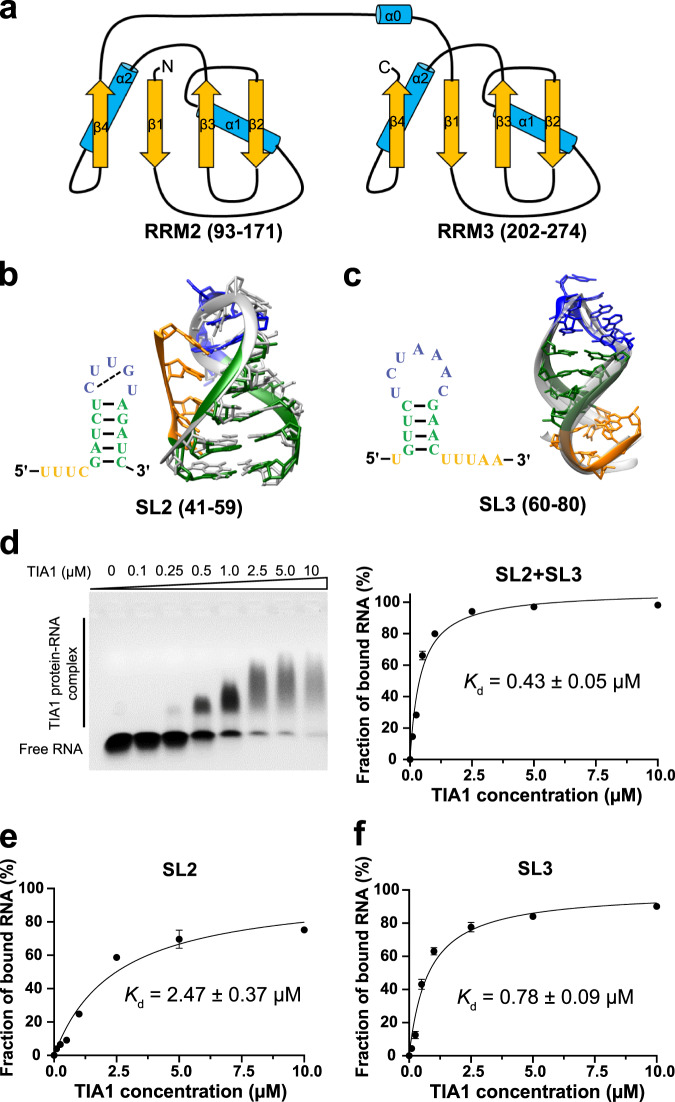
Structures of SARS-CoV-2 RNA genome stem-loop (SL) SL2 and SL3 elements and their binding ability to human TIA1 protein. **a** Architecture of TIA1 RNA recognition motif (RRM) RRM2 and RRM3 domains. RRM2 and RRM3 adopt the canonical βαββαβ RRM fold composed of four anti-parallel β-sheets (β1, β2, β3, and β4) and two α-helices (α1 and α2). A non-canonical helix α0 in the flexible linker is preceded to RRM3. Secondary and 3D structures for (**b**) SL2 and (**c**) SL3 RNA elements. Stem, hairpin loop, and terminal loop in 5′-/3′-end are colored by green, blue, and orange, respectively. All 3D structures are predicted by IsRNA2 model. The NMR solution structure (1st model) for SARS-CoV SL2^32^ (PDB id: 2l6i) is given in gray in (**b**). Other possible 3D conformations for SL3 element predicted by IsRNA2 are shown in light gray in (**c**). Positions from the whole SARS-CoV-2 RNA genome for SL2 and SL3 elements are provided in parentheses. **d** EMSA results (left) and the binding curve (right) show TIA1 bound to 5′ end Cy3-labeled SL2 + SL3 RNA. The binding curves for individual (**e**) SL2 and (**f**) SL3 RNA element with TIA1 protein. From **d** to **f**, the *K*_d_ values were calculated from the EMSA image quantification from three independent experiments. Data are presented as mean ± SD. Source data are provided as a Source Data file.

TIA1 is a ubiquitous RBP^20^ that plays multifunctional regulatory roles in the gene expression layers, such as transcription, alternative splicing, and translation of mRNAs, and in the physio(patho)logical-associated events, such as cell stress, apoptosis, viral infections, etc^21^. TIA1 contains three N-terminal RNA recognition motifs (RRMs) connected by flexible linkers and interacts mainly with the target RNA segments via the second and third RRMs^22,23^. Structural studies^23,24^ indicated that both RRM2 and RRM3 adopt the canonical βαββαβ RRM fold composed of a four-stranded anti-parallel β-sheet covered on one side by two α-helices (see Fig. 1a). Previous works have shown that TIA1 and its related protein TIAR could interact with West Nile^25–27^, dengue^25^, and tick-borne encephalitis^28^ virus’ RNAs and play vital roles in their life cycle. More specifically, Zhang and coworkers^18^ recently predicted that TIA1 could bind to the SL2/3 element of the SARS-CoV-2 RNA genome by using a deep-learning tool based on the in vivo RNA structural data. The binding of TIA1 to SARS-CoV-2 viral RNA was validated by RNA pull-down assay subsequently^18^ and was also confirmed in an another independent experiment through ChIRP-MS method^7^. Moreover, they found that both antisense oligonucleotides (ASOs) targeting the SL2/3 RNA element and efficient depletion of the TIA1 protein by siRNA knocking down could dramatically reduce SARS-CoV-2 infects in human cells. Thus, those points further emphasize the vital function role of cis-acting RNA elements (such as SL2/3) and their cognate host RBPs (such as TIA1) in the SARS-CoV-2 life cycle. However, the molecular mechanism and structural details of the binding between the SL2/3 RNA element of SARS-CoV-2 and host TIA1 protein remain unclear, which largely hinders further efforts to develop desirable RNA-targeting antiviral drugs.

As a complement to expensive experimental structural characterizations, computational predictions for the 3D structures of cis-acting RNA elements and their binding complexes with host proteins could provide adequate information for virtual screening of novel RNA-targeting antivirals before accurate but time-consuming determination of related atomic details. For instance, computational models for 5′-UTR regions have been used for in silico ligand screening to inhibit SARS-CoV-2 replication^29^. In another prior work by Park et al., a 3D structural model of the SARS-pseudoknot was constructed, then a novel ligand that dramatically inhibits the −1 ribosomal frameshifting of SARS-CoV has been identified by structure-based virtual screening^30^. Here, combined with molecular modeling, free energy calculations, electrophoretic mobility shift assays (EMSA) and in vivo ASO assays, we identified the binding mode of the SL3 RNA element and host TIA1 protein, constructed the putative 3D structure for the binding complex and evaluated various mutagenic point mutations on the key nucleotides (nts) at the binding interface. We expected those knowledges could provide new insights into viral mechanisms during infection and lay down the foundation for effective RNA-targeting antiviral drug design.

## Results

### Structures of SL2 and SL3 RNA elements

Previous studies^13–19^ have demonstrated the secondary structures of SARS-CoV-2 SL2 and SL3 RNA elements and their ability to fold independently. As shown in Fig. 1b, c, both SL2 and SL3 elements adopt the hairpin structure with a flanked tail at 5′- and/or 3′-end. Then, we employed the IsRNA2 model^31^ to predict 3D structures of SL2 and SL3 RNA elements. Our previous work indicated that the coarse-grained IsRNA2 model enables de novo modeling RNA 3D structures with a comparable performance to the atomic model but at much less cost^31^. For the SL2 element, the predicted 3D structure adopts nearly the same global fold as the NMR solution structure of SARS-CoV SL2^32^ (Protein Data Bank (PDB) id: 2l6i), which shares the identical sequence with SARS-CoV-2 SL2, and the heavy-atom root-mean-square deviation (RMSD) between two structures is 2.2 Å (see Fig. 1b). These points further declare the capability of IsRNA2 model in RNA 3D structure prediction. For the SL3 segment, the predicted 3D structure indicates that five (A68, A69, A70, C71, and G72) nts in the hairpin loop (HP) trend to form consecutive base stackings, while the 3′-end 5-nt terminal loop (TL) is somewhat flexible. Recently, the 3D structure of SL3 RNA element was also predicted by FARFAR2^33^ and all-atom MD simulation^34^. Our IsRNA2 predicted structure shares a similar fold to FARFAR2 with heavy-atom RMSD of 1.4 Å, but is slightly different from the prediction via all-atom MD in the HP region (see Supplementary Fig. 1).

### TIA1 RRM2_3 mainly binds to SL3 RNA element

RRMs are the most abundant RNA binding motif and are well known for their ability to bind most commonly 3- to 5-nt stretches of single-stranded oligonucleotide^35^ (linear stretches or loop regions). The RRMs of a single protein can contribute differentially to the overall RNA binding, in terms of both affinity and specificity. For TIA1 protein, early experiments^22,24^ indicated that its preferred target is U-rich sequences predominantly directed by RRM2. Its RRM1 is thought to have little intrinsic RNA binding affinity and contribute trivially to RNA binding in the context of RRM1,2,3, while the RRM2,3 may bind cooperatively to pyrimidine-rich RNA sequences^23^. To explore the interactions between SARS-CoV-2 SL2/3 RNA elements and human TIA1 protein in detail, we purified recombinant TIA1 (Supplementary Fig. 2) and performed EMSA experiments. Expectedly, compared with the TIA1 RRM1-3 protein (1–274aa), the TIA1 RRM2_3 truncation (93–274aa, Fig. 1a) shows similar binding ability with both SL2 + SL3 and U11^23^ (positive control) RNA sequences (see Fig. 1d and Supplementary Fig. 3). In contrast, the negative control oligoC (C21) RNA segment only displays a trivial binding ability to TIA1 (Supplementary Fig. 4), which agrees with the previous study^26^. For simplicity, we focus on the TIA1 RRM2_3 truncation in the following sections and treat it as the TIA1 protein.

To further dissect the major binding site of TIA1, we measured the dissociation constants (*K*_d_) of TIA1 with SL2 + SL3, SL2 and SL3 from EMSA experiments, respectively. The *K*_d_ of TIA1 with full SL2 + SL3 is 0.43 ± 0.05 µM, which is consistent with published *K*_d_ values of TIA1 with other oligonucleotide substrates^23,24^ (Fig. 1d and Supplementary Fig. 5). For individual SL RNA elements, the *K*_d_ of TIA1 with SL3 is slightly increased (*K*_d_ = 0.78 ± 0.09 µM), but that for SL2 is dramatically increased to 2.47 ± 0.37 µM (Fig. 1e, f). That is to say, the binding abilities of SL2 + SL3 and isolated SL3 element to TIA1 protein are comparable, while the binding of SL2 is obviously weaker than the others. The differences in sequences and length of HP (5-nt in SL2 vs. 7-nt in SL3, Fig. 1b, c) may account for the weaker binding of SL2 relative to SL3 RNA element. Overall, the SARS-CoV-2 SL3 RNA element serves as the major binding site for human TIA1 protein.

### Both HP and 3′-TL of SL3 element are essential for TIA1 binding

**Fig. 2:**
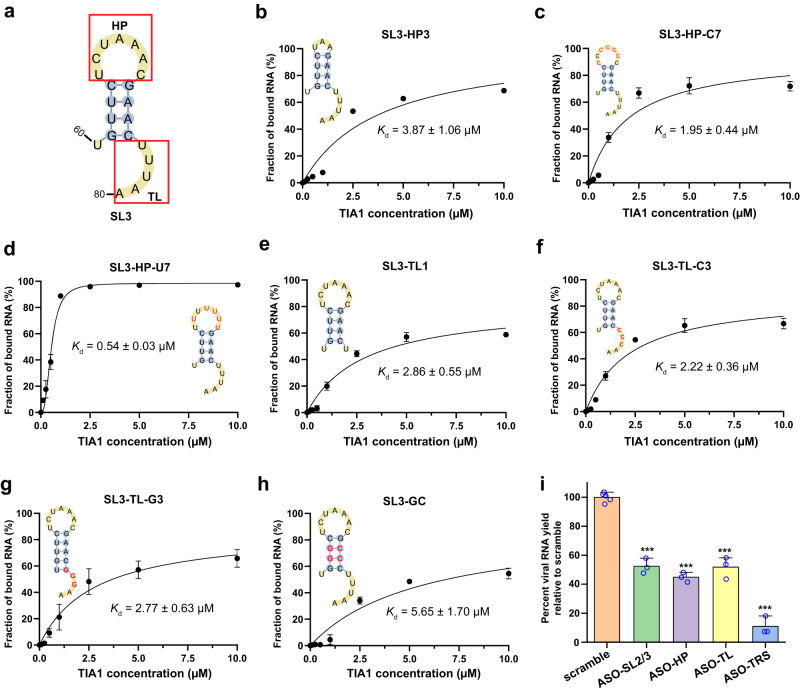
Binding capabilities of various SL3 RNA variants with human TIA1 protein. **a** WT SL3 consists of a 7-nt hairpin loop (HP), a 4-base pairs stem, and a 5-nt 3′-terminal loop (TL). The binding curves of human TIA1 protein with various mutated and truncated variants of SL3 RNA element: **b** SL3-HP3, the 7-nt HP reduced to 3-nt length, **c** SL3-HP-C7, sequence of 7-nt HP mutated to oligo C, (**d**) SL3-HP-U7, sequence of 7-nt HP mutated to oligo U, **e** SL3-TL1, last four nucleotides of 5-nt TL deleted, **f** SL3-TL-C3, three successive uridines in TL replaced by three cytosines, **g** SL3-TL-G3, three successive uridines in TL replaced by three guanines, **h** SL3-GC, two middle A-U base pairs in the stem substituted by G-C base pairs. All the secondary structures for SL3 variants are predicted by RNAStructure^36^ and mutated nucleotides are colored by red. All the *K*_d_ values were calculated from the EMSA image quantification from three independent experiments. Data are presented as mean ± SD. **i** The yield of the bona fide SARS-CoV-2 virus with designed ASOs targeting SL3 RNA elements (including HP and TL loops and the transcriptional regulatory sequence (TRS)) in Huh7.5.1 cells for 24 h, compared to the “scramble” control treated with a non-targeting ASO. SL2/3 has previously been reported^18^. Data represent the mean ± SEM; *n* = 3 biological replicates. ^***^p = 3.4 × 10^−6^ (ASO-SL2/3), 4.4 × 10^−7^ (ASO-HP), 1.2 × 10^−5^ (ASO-TL), and 1.6 × 10^−7^ (ASO-TRS) using unpaired two-sample Student’s *t* test. Source data are provided as a Source Data file.

In order to determine the binding mode of SL3 RNA element with human TIA1 protein, we measured the binding affinities for various mutated and truncated variants of SL3. The SARS-CoV-2 SL3 RNA element folds into a SL structure that contains a 7-nt HP, a stem consisting of four base pairs and a 5-nt 3′-TL (Fig. 2a). Since RRM commonly recognizes 3- to 5-nt stretches of single-stranded oligonucleotide, the roles of HP and TL in TIA1 binding were evaluated individually. Firstly, when the 7-nt HP was truncated to a 3-nt loop (named SL3-HP3, Fig. 2b), we found that the binding affinity of TIA1 is significant decreased (*K*_d_ = 3.87 ± 1.06 µM), demonstrating the HP is required for TIA1 binding. Moreover, the 7-nt HP was mutated to all cytosines (SL3-C7) or uridines (SL3-U7) to check sequence preference. EMSA results showed SL3-C7 variant has a lower binding affinity (*K*_d_ = 1.95 ± 0.44 µM) but SL3-U7 variant has a higher binding affinity with TIA1 (*K*_d_ = 0.54 ± 0.03 µM) than wild-type SL3 (Figs. 1f and 2c, d). This scenario is consistent with binding character of TIA1 that prefers U-rich element^22–24^ (Supplementary Fig. 3) and disfavors all C’s loop^26^ (Supplementary Fig. 4).

Subsequently, the function of 5-nt 3′-TL for TIA1 binding was assessed. As shown in Fig. 2e, deletion of that TL from SL3 (SL3-TL1) obviously decreases the binding affinity with TIA1 (*K*_d_ = 2.86 ± 0.55 µM), indicating the 5-nt TL is also essential for TIA1 binding. Considering that three successive uridines (U76, U77, and U78) are present in the TL and the strong U-rich preference of RRM2^22–24^, we speculated that SL3 interacts with TIA1 RRM2 through those three uridines. To validate this assumption, we replaced those 3 Us to 3 Cs (SL3-TL-C3) or 3 Gs (SL3-TL-G3) and repeated the measurements of their binding ability with TIA1. Expectedly, either SL3-TL-C3 or SL3-TL-G3 variant has a lower binding affinity than WT SL3 (Figs. 1f and 2f, g). Taken together, both the 7-nt HP and the 5-nt 3′-TL of SL3 RNA element interact with TIA1 protein and the putative binding mode may be that the HP binds with RRM3 and the 3′-TL interacts with RRM2.

In addition, the impact of stability of the stem on binding was also explored. The stem of SL3 consists of two middle A-U base pairs and two terminal G-C base pairs. Due to the higher stability of G-C base pair over A-U one, the substitution of those two A-U base pairs with G-C ones (SL3-GC, see Fig. 2h) should increase the stability of SL3. Indeed, the folding free energy change predicted by RNAStructure^36^ is increased from −2.1 kcal/mol (SL3) to −6.6 kcal/mol for SL3-GC. Intriguingly, superior stability of the stem inhibits the TIA1 binding and results in a weaker binding affinity *K*_d_ = 5.65 ± 1.70 µM (Fig. 2h). Therefore, a relatively loose stem structure is required for SL3 RNA element binding with TIA1 protein, probably to facilitate the RNA structure rearrangement during TIA1’s binding to both sites (HP and 3′-TL) of SL3.

To further validate the functional impact of SL3 element, several ASOs were designed to perturb the aforementioned interactions between SL3 and TIA1 protein. Specifically, five ASOs with a 2′-O-methoxyethyl and a phosphorothioate backbone modification were synthesized (see Supplementary Fig. 6 and Supplementary Table 1), including a non-targeting scramble ASO (negative control), the ASO-SL2/3 from previous study^18^ (positive control), an ASO-HP targeting the HP, an ASO-TL targeting the 3′-TL, and an ASO-TRS targeting the transcriptional regulatory sequences^37^ (TRS). In agreement with the previous study^18^, we observed decreased SARS-CoV-2 RNA yield (to ~52.4%) in Huh7.5.1 cells (human liver cancer cell line) transfected with ASO-SL2/3 in comparison with the scramble control (see Fig. 2i and Supplementary Table 2). Then, disruptions of interactions between SL3 RNA element and TIA1 protein through ASO-HP and ASO-TL resulted in ~55.1% and 48.2% decreased viral RNA yields, respectively. Notably, transfected with ASO-TRS could largely decrease SARS-CoV-2 RNA yield (to ~10.9%) due to the critical role of TRS in viral RNA replication^37^. All the ASOs did not show significant cytotoxicity (Supplementary Fig. 6 and Supplementary Table 2). Together, those data declared that both the HP and 3′-TL of SL3 RNA element are functionally important in SARS-CoV-2 life cycle and the interactions between SL3 and TIA1 protein may be crucial.

### Putative 3D model for SL3 and TIA1 complex

**Fig. 3:**
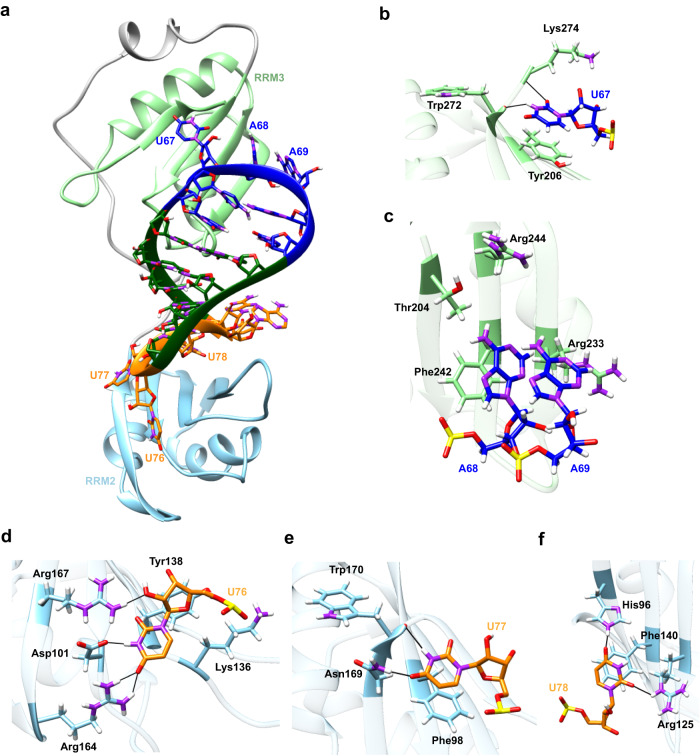
The putative 3D binding model for SARS-CoV-2 SL3 RNA element and human TIA1 complex. **a** Overview of the most probable 3D structure of binding complex constructed by computational modeling. TIA1 RRM2, RRM3, and flexible linker are colored by sky blue, light green, and gray, respectively. Hairpin loop, stem, and terminal loop of SL3 are separately colored by blue, green, and orange. Details of interactions between TIA1 RRM3 and nucleotides (**b**) U67, (**c**) A68 and A69 in hairpin loop region of SL3 element. Details of interactions between TIA1 RRM2 and nucleotides (**d**) U76, (**e**) U77, and (**f**) U78 in 3′-terminal loop of SL3. Atoms of phosphorus, oxygen, nitrogen, and hydrogen are colored yellow, red, purple, and white, respectively, and carbon atoms are colored according to their locations. Hydrogen bonds between RNA nucleotides and TIA1 residues are indicated by thin black lines. Rotations may be adopted to display the interaction details in subfigures (**b**–**f**).

Based on the above knowledge, the 3D binding model for SARS-CoV-2 SL3 RNA element with human TIA1 protein was constructed computationally through template-based approach and MD simulations. The entire procedure of computational modeling contains four steps and the details are given in Method and Materials section. Apart from the most probable conformation displayed in Fig. 3, other possible 3D models of the binding complex extracted from the 2.5-μs MD simulations (see Supplementary Fig. 7) are displayed in Supplementary Fig. 8 and Supplementary Data 1. The superior stability of the putative 3D binding model was validated by three independent 1-μs MD simulations, in which stable heavy-atom RMSD values (~2.5 Å) and potential energies between RNA and protein (~1762 kJ/mol) are observed during all three simulations (Supplementary Fig. 9). Furthermore, similar binding interfaces relative to the selected templates^24,38^ remained intact after long MD simulations (Supplementary Fig. 10). In contrast, the binding model for all C’s loop variant of SL3, which has a much weaker binding ability to TIA1 protein^26^, seems unstable under the same simulation conditions, which is characterized by larger fluctuation of RMSD and less number of contacts between RNA and protein (see Supplementary Fig. 11). In agreement with the previous study^24^, SL3 binding induces a compact domain arrangement for TIA1 protein, which is highly flexible in its apo state^23^, and the RRM2 and RRM3 domains cooperate in binding to SL3 RNA element (Fig. 3a). For SL3 element, compared to the free state (Fig. 1c), binding to TIA1 obviously stretches both the hairpin and 3′-TLs and the heavy-atom RMSD between predicted free and bound structures is 4.9 Å (see Supplementary Fig. 12). Thus, SL3 binding by TIA1 causes notable structural changes both in protein domain arrangement and RNA 3D structure adaptation.

Since the β-sheet surfaces of the TIA1 RRM2 and RRM3 serve as the classical oligonucleotide binding interface^39^, lots of positively charged residues, such as Arg125, Lys136, and Arg167 in RRM2 and Arg233, Lys238, and Lys274 in RRM3, are resided on the sheet surfaces to accommodate the negatively charged RNA backbone. For the base moieties in the binding interface, the main interactions include aromatic stacking, specific hydrogen bonds (H-bonds), as well as hydrophobic interactions (Fig. 3 and Supplementary Table 3). Specifically, four conserved aromatic residues (Phe98 and Phe140 in RMM2 and Tyr206 and Phe242 in RRM3^24^) located in the RNP-2 and RNP-1 sub motifs of RRMs interact directly with RNA bases U77, U78, U67, and A68 through π–π stacking, respectively. Additionally, U67 forms two H-bonds via N3–H3 and O2 atoms with Trp272 and Lys274, respectively (see Fig. 3b and Supplementary Table 3). And an acyclic stacking interaction between U67 and Lys274 is present. For other nts in the HP region, A68 and A69 possess rich van der Waals (vdW) interactions with residues from TIA1 RRM3 (Fig. 3c). In the case of the 3′-TL, U76 forms four H-bonds through atoms O2, N3–H3, O4, and O2’ (Fig. 3d and Supplementary Table 3), U77 forms two H-bonds via N3–H3 and O4 atoms (Fig. 3e and Supplementary Table 3), and U78 forms two H-bonds through atoms O2 and O4 (Fig. 3f and Supplementary Table 3), respectively. It should be noted that the N3–H3 and O4 atoms involved in above H-bonds are unique for uracil and could not be taken place by other RNA bases, which may facilitate to understand the U-rich preference for TIA1 binding.

As the sequences of TIA1 and its related protein TIAR are highly similar (sequence identity 89%) and the identified residues that interact with SL3 RNA element are all conserved (see Supplementary Fig. 13), the 3D binding model for SL3 and TIAR was also constructed through sequence replacement and subsequent MD simulations. In general, the SL3 and TIAR binding complex adopts a similar global fold with SL3 & TIA1 (Supplementary Fig. 13), and all the key interactions identified above between RNA and protein were reserved in the course of MD simulations (see Supplementary Table 3). Thus, it is plausible that TIA1 and TIAR interact with SARS-CoV-2 SL3 RNA element in a nearly identical manner.

### Influence of single nucleotide mutations on SL3 binding

**Fig. 4:**
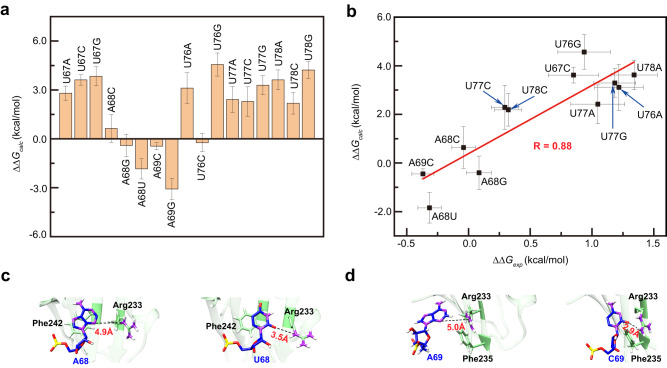
Influence of single nucleotide mutations of SL3 RNA element on TIA1 binding. **a** The relative binding free energy changes $\Delta \Delta {G}_{{\rm calc}}$ for exhaustive single nucleotide mutations of six identified sites that are important for SL3 binding. $\Delta \Delta {G}_{{\rm calc}} \, < \, 0$ means enhanced binding affinity with TIA1 protein than wild type SL3 element, and vice versa. Five independent FEP calculations were run and data are presented as mean ± SD. **b** Comparison of the relative binding free energy changes derived from FEP calculations ($\Delta \Delta {G}_{{\rm calc}}$) and EMSA experiments ($\Delta \Delta {G}_{\exp }$) for twelve representative mutations of SL3. The linear fit to the data is shown as a red line and $R$ is the Pearson correlation coefficient. Five independent FEP calculations were run for $\Delta \Delta {G}_{{\rm calc}}$ and data are presented as mean ± SD. The values of $\Delta \Delta {G}_{\exp }$ were derived from three independent EMSA experiments and data are presented as mean ± SD. Source data are provided as a Source Data file. Structural details before (left) and after (right) mutations for SL3 (**c**) A68U and (**d**) A69C variants. The phosphorus, oxygen, nitrogen, and hydrogen atoms are colored by yellow, red, purple, and white, respectively. The carbon atoms in SL3 and TIA1 are separately colored to blue and light green. Black dash lines indicate the distances of given atom pairs.

The putative 3D structure of SL3 and TIA1 binding complex proposed above suggests that six nts (U67, A68, A69, U76, U77, and U78) play important roles in directing SL3 binding to TIA1 protein. Therefore, exhaustive mutations were introduced for each of those six nts and the relative binding free energy changes (ΔΔ*G*_calc_) were estimated through free energy perturbation (FEP) calculations. Up to now, FEP is regarded as the most rigorous and reliable method in estimating binding affinity changes, which has also achieved high accuracy in characterizing vital residues and their mutational effects for many protein–protein, protein–ligand, and protein–DNA bindings, as compared with experiments^40–45^. As shown in Fig. 4a and Supplementary Table 4, mutations in U67 site are all adverse, e.g., ΔΔ*G*_calc_ = 3.62 ± 0.33 kcal/mol for U67C and ΔΔ*G*_calc_ = 3.84 ± 0.58 kcal/mol for U67G mutations, and are predicted to decrease the binding affinities. Likewise, mutations for nts U76 (except for U76C), U77, and U78 are also all unfavorable, such as U77 (U78) with the least binding free energy change ΔΔ*G*_calc_ = 2.29 ± 0.91 kcal/mol (ΔΔ*G*_calc_ = 2.18 ± 0.66 kcal/mol) at U77C (U78C) mutation. The aforementioned U’s particular H-bonds may partially account for those disfavors, including U67@N3–H3…Trp272@O (Fig. 3b), U76@N3–H3…Asp101@Oδ1 (Fig. 3d), Asn169@Nδ2–Hδ2…U77@O4 (Fig. 3e), and so on. Those results reprove the U-rich binding preference of TIA1 protein. However, impacts of mutations at A68 and A69 sites are diverged (see Fig. 4a and Supplementary Table 4). On one hand, A68C (ΔΔ*G*_calc_ = 0.64 ± 0.86 kcal/mol) and A68G (ΔΔ*G*_calc_ = −0.40 ± 0.68 kcal/mol) were predicted to have trivial influence on binding affinity. On the other hand, the predicted binding free energy changes for A68U and A69G mutations are ΔΔ*G*_calc_ = −1.84 ± 0.63 and −3.07 ± 0.65 kcal/mol, respectively, which indicates an enhanced binding affinity between those two SL3 variants and TIA1 protein.

To further validate the influence of single nucleotide mutations indicated by FEP approach, the binding affinities (*K*_d_) for some representative mutations were measured from ESMA experiments (see Supplementary Fig. 14). For comparison, the experimental binding free energy changes (Supplementary Table 4) are derived as $\Delta \Delta {G}_{\exp }=-{k}_{{{{{\rm{B}}}}}}T{{{{{\rm{ln}}}}}}({K}_{{{{{\rm{d}}}}}}^{{{{{{\rm{wt}}}}}}}/{K}_{{{{{\rm{d}}}}}}^{{{{{{\rm{mut}}}}}}})$, where ${K}_{{{{{\rm{d}}}}}}^{{{{{{\rm{wt}}}}}}}$ and ${K}_{{{{{\rm{d}}}}}}^{{{{{{\rm{mut}}}}}}}$ are dissociation constants for wild type and mutated SL3 elements, respectively, and ${k}_{{{{{\rm{B}}}}}}$ is the Boltzmann constant and $T=300{{{{{\rm{K}}}}}}$. For twelve selected mutations (at least one case for each of aforementioned six key sites for SL3 binding), a strong correlation with Pearson coefficient $R=0.88$ between experimental ($\Delta \Delta {G}_{\exp }$) and calculated (ΔΔ*G*_calc_) binding free energy changes is observed (see Fig. 4b). Although the magnitude of the binding affinity changes is generally larger in FEP calculations, this is probably due to the imperfect force field parameters yet. This point declares that those elect six nts (U67, A68, A69, U76, U77, and U78) are indeed important for SL3 binding by TIA1 protein and indirectly prove the reliability of the 3D binding model proposed in Fig. 3.

Particularly, both the FEP calculation and subsequent experiment indicate SL3 A68U (*K*_d_ = 0.46 ± 0.05 µM) and A69C (*K*_d_ = 0.42 ± 0.04 µM) variants have an enhanced binding affinity to TIA1 protein than the wild type (*K*_d_ = 0.78 ± 0.09 µM). Inspection of the detailed interactions before and after mutations in MD simulations reveal that an extra H-bond was formed between side chain of Arg233 at TIA1 RRM3 and SL3 U68/C69 variant (Fig. 4c, d), which is indicated by the decreased pair distances (such as A68@N3–Arg233@Nε vs. U68@O2–Arg233@Nε in Supplementary Fig. 15) and increased occupancies of particular interactions (such as 4.5% for Arg233@Nη-Hη…A69@N3 vs. 45.4% for Arg233@Nη–Hη…C69@O2 in Supplementary Table 4). Considering the potentially vital function of SL3-TIA1 binding in SARS-CoV-2 replication, A68U and A69C can be treated as possible variants of concern for COVID-19.

### Interactions between TIA1 protein and SL3 RNA elements are common for betacoronavirus genomes

**Fig. 5:**
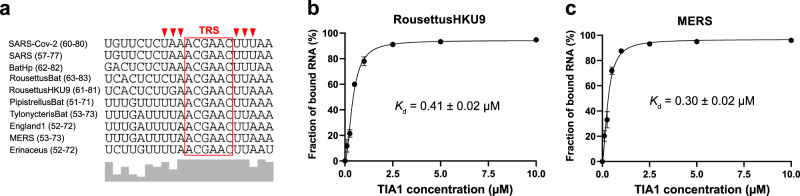
Sequence comparison of SL3 RNA elements in betacoronavirus genomes and their binding abilities to human TIA1 protein. **a** Multiple sequence alignment showing nucleotide coverage within SL3 RNA elements of representative species within genus *Betacoronavirus*. The transcriptional regulatory sequences (TRS) are highlighted by red box. Six identified sites important for SARS-CoV-2 SL3 binding by TIA1 protein are marked by red triangles. Sequence positions of SL3 RNA element in each species are given in parentheses. BatHp, Bat Hp-betacoronavirus/Zhejiang2013; RousettusBat, Rousettus bat coronavirus; RousettusBatHKU9, Rousettus bat coronavirus HKU9; PipistrellusBat, Pipistrellus bat coronavirus HKU5; TylonycterisBat, Tylonycteris bat coronavirus HKU4; England1, Betacoronavirus England 1; MERS, Middle East respiratory syndrome-related coronavirus; Erinaceus, Betacoronavirus Erinaceus/VMC/DEU/2012. The binding curves between human TIA1 protein and SL3 RNA elements from (**b**) RousettusBatHKU9 and (**c**) MERS genomes. The *K*_d_ values were calculated from the EMSA image quantification from three independent experiments. Data are presented as mean ± SD. Source data are provided as a Source Data file.

Apart from the SARS-CoV-2, the other members of betacoronavirus also cause illness in humans and animals, including the SARS coronavirus that caused the SARS outbreak in 2003^46^ and the Middle East respiratory syndrome (MERS) coronavirus that triggered the MERS outbreak in 2012^47^. In addition to the identical SL3 shares by SARS-CoV-2 and SARS genomes, multiple sequence alignment^5,11,12^ indicates that SL3 RNA elements are well conserved among different species within genus *Betacoronavirus* (Fig. 5a). More specifically, the two identified binding cores in SARS-CoV-2 SL3 RNA element to TIA1 protein are highly conserved, namely, the 5′-U[A/U]A-3′ and 5′-UU[U/A]−3′ segments located before and after the TRS^37^, respectively. Thus, we assumed that TIA1 protein could interact with other SL3 RNA elements within betacoronavirus genomes. To validate this hypothesis, the binding abilities of two representative SL3 RNA elements from other members of betacoronavirus to human TIA1 protein were studied by EMSA experiments. Expectedly, high binding affinities with *K*_d_ = 0.41 ± 0.02 µM (Rousettus bat coronavirus HKU9) and *K*_d_ = 0.30 ± 0.02 µM (MERS) were observed (Fig. 5b, c) for those two different SL3 RNA elements. Furthermore, consisting with the higher binding affinity for the A68U SL3 variant of SARS-CoV-2 (Fig. 4), six of ten concerned betacoronavirus genomes adopt uridines in the corresponding position and only three members adopt adenines, which results in the 5′-U[U/A]A-3′ binding motif preceded to the TRS (Fig. 5a). Overall, interactions between the SL3 RNA elements of betacoronavirus genomes and human TIA1 protein are common. We speculated that this viral RNA-host protein interaction plays an indispensable role in the life cycle of betacoronavirus.

## Discussion

RNA-protein interactions are pervasive in biology and play numerous roles in cellular function and disease^48,49^. Due to the critical relationships between structure and function, structural and mutagenesis studies of RNA-RBP interactions are highly desired. However, only several hundreds of RNA/RBP complexes can be found among the ~4400 experimentally determined structures of protein-RNA complexes currently deposited in the PDB database. The paucity of RNA/RBP structures mainly comes from the inherent difficulties in the crystallization of protein–RNA complexes that are most likely linked to the high flexibility of RNA molecules. In this scenario, the emerging computational modeling is very attractive, which largely benefits from the recent rapid advancements in protein^50,51^ and RNA 3D modeling^52–54^, ever improving atomic force fields for protein and RNA molecules^55–58^, and the ready availability of high-performance computing.

Particularly, though interactions between viral RNA and host RBPs are indispensable during RNA virus transcription and replication, which RBPs and how they interact with viral RNA genomes remain largely unclear. Here, we systematically investigated the binding interactions between the SARS-CoV-2 cis-acting RNA elements and human TIA1 protein through a combined theoretical and experimental approach including molecular modeling, free energy calculations, EMSA, and ASO assays. To the best of our knowledge, this is the first time that a detailed study for the binding interface between the cis-acting element in the 5′-UTR of SARS-CoV-2 RNA genome and a particular human protein (namely TIA1) was performed. We first confirmed that the SL3 element of SARS-CoV-2 RNA genome can bind to TIA1 protein RRM2_3 truncation with high affinity (*K*_d_ = 0.78 ± 0.09 µM). Then, both the 7-nt HP and 5-nt 3′-TL were found to be essential for SL3 in its binding with TIA1 protein in a sequence-dependent manner, in which truncations of loops or sequence alterations in loop regions by cytosines/guanines obviously decrease the binding affinity. And disruptions of the SL3 RNA and TIA1 binding with specific ASOs designed apparently decreased the bona fide SARS-CoV-2 RNA yields in Huh7.5.1 cells. After that, a 3D model for the binding complex between SL3 RNA element and TIA1 protein was constructed by template-based approach and MD simulations. Along with the electrostatic interactions between positively charged residues and the negatively charged RNA backbone, the aromatic stacking, specific H-bonds, and hydrophobic interactions collectively direct the specific binding of SL3 to TIA1 protein. Moreover, for six identified nts (U67, A68, A69, U76, U77, and U78) at the binding interface, exhaustive single nucleotide mutations were introduced (in silico mutagenesis) and their relative binding free energy changes were calculated. Specifically, FEP calculation and subsequent experiments demonstrated that the SL3 A68U (~1.7-fold) and A69C (~1.9-fold) variants have an enhanced binding affinity to TIA1 protein than the wild type. Finally, due to sequence conservation of the cis-acting elements, we found that the human TIA1 protein also interacts with other SL3 elements from betacoronavirus RNA genomes, such as the MERS and SARS coronavirus, in comparable binding affinities. In conclusion, those presented data provide a pioneering structural basis to understand the viral RNA-host protein interactions for SARS-CoV-2 (which might be extended to other betacoronavirus infections).

Due to the RNA nature of its genome, SARS-CoV-2 can rapidly evolve and adapt towards vaccines and drugs by slightly altering their core protein-coding sequences^59^, such as those encoding for the spike protein. Thus, no vaccine has been proven to have sustained efficacy against the recently reported SARS-CoV-2 variants, such as the Omicron variant, which highlights the need for development of novel and synergistic antivirals^60^. In contrast to the highly mutated protein-coding regions, the cis-acting elements in the UTR of SARS-CoV-2 are much more stable (of ~10^−5^ mutation rate in 1,147,000 variants) and are associated with the regulation of viral replication, subgenomic mRNA production and translation. Therefore, development of novel antivirals targeting those cis-acting RNA elements is particularly attractive and several pioneering works have been performed recently^29,61,62^. To this end, our study here and previous works^33,34,63^ provided putative 3D structural information for future structure-based drug designs. For instance, since SL3 element binding to TIA1 protein requires notable conformation changes both in protein domain arrangement and RNA 3D structure adaptation, drug-like compounds that either disrupt RNA 3D structure adaptations or interfere with the viral RNA-host protein interactions are promising.

## Methods

### 3D structure predictions of SL RNA elements

The IsRNA2 model^31^ was employed to predict the 3D structures for SARS-CoV-2 SL2 and SL3 RNA elements. With the sequences and secondary structures of SL2 and SL3 elements (Fig. 1b, c) as inputs, a procedure identical to previous study^31,64^ was utilized to obtain the predicted 3D RNA structures. In brief, initial 3D structures were generated firstly by Vfold3D^65^/VfoldLA^66^ programs from input. Then, 50 ns replica-exchange molecular dynamics (MD) simulations with 10 replicas possessing temperatures from 200 to 425 K were run in coarse-grained representations to sample the 3D conformational space. After that, a clustering process based on the top 10% snapshots with the lowest potential energies was used to obtain the candidate structures. Finally, the centroid structure of the largest cluster followed by all-atom reconstruction and energy minimization presented the predicted 3D conformation.

### Molecular dynamics simulations

All-atom MD simulations were conducted using Gromacs^67^ (version 2020.6) to further relax the predicted 3D structures. For each simulated system, a water box with at least 1.5 nm distant from the surface of the RNA or RNA & TIA1 complex was used to solvate the systems with 50 mM NaCl ions added. The Amberff14SB^57^ force field was used for protein and RNA^58^ parameters. Water molecules were described by TIP3P model^68^ and Li and Merz 12-6 ion parameters^69^ were used. The periodic boundary conditions were applied in all three dimensions. The particle mesh Ewald (PME) method was used to compute the long-range electrostatic interactions while the vdW interactions were truncated at 1.5 nm. The LINCS algorithm^70^ was adopted to constrain the H-bonds to allow an integration timestep of 2 fs. Before MD productions, an energy minimization, 100 ps NVT, and 1 ns NPT simulation with a temperature of *T* = 300 K and pressure of 1 atm was executed sequentially to equilibrate the simulation box. To avoid unexpected structural deviations in the beginning, a further 15 ns MD simulation with gradually weakening position restraints on C1′ atoms of RNA molecule and Cα atoms of protein was subsequently performed in the NPT ensemble. After that, series of MD simulations (1000/1500/2500 ns) for isolated RNA and RNA&TIA1 complex were conducted in the NPT ensemble with velocity-rescaled Berendsen thermostat^71^. Three duplicate simulations were run for each concerned system. A summary of the simulation systems performed in this study was given in Supplementary Table 5. Snapshots extracted from the last 500 ns MD trajectories (2500 snapshots recorded in the duration of 200 ps) were clustered based on gromos method^72^ with a 0.3 nm RMSD cutoff to obtain the most probable conformation. The analysis of occupancies of key interactions between RNA and protein was done by VMD^73^. The 3D structure models were rendered by UCSF Chimera^74^ programs.

### Construction of the binding complex

We combined template-based approach with MD simulations to construct the 3D structure of the binding complex between SL3 RNA element and TIA1 RRM2_3 truncation. The entire procedure contains four main steps. The first step is to find suitable template for individual RRM and its bound RNA fragment from PDB. Since the 3D structure for TIA1 RRM2 recognition of target oligonucleotide (5′-TTT-3′) has been determined by X-ray diffraction^24^ (PDB id: 5ith), the binding complex between RNA fragment 76UUU78 and RRM2 was achieved straightforward through replacing the thymine by uracil. For complex of RRM3 and HP of SL3 element, we selected the crystal structure of the RNA-binding domain of U1A spliceosomal protein complexed with an RNA hairpin as a template^38^ (PDB id: 1urn). Due to highly structural similarity between different canonical RRMs, the TIA1 RRM3 extracted from the apo state^23^ (PDB id: 2mjn) could consistently align to U1A protein with RMSD 1.7 Å over Cα atoms. Then, the RNA segment 9GCAC12 in the U1A/RNA complex was chosen as a feasible template for fragment 66CUAA69 of SL3 RNA in the binding complex. The second step is to predict the possible 3D structures for SL3 RNA element in its bound form. In addition to sequence and secondary structure (see Fig. 1c) as inputs, the templates for fragments 66CUAA69 and 76UUU78 were also introduced as rigid constraints in IsRNA2 model^31^. Following the same process mentioned above for free SL RNA 3D structure prediction, we obtained two possible 3D bound conformation for SL3 RNA element. The third step is to construct the initial binding complex for SL3 RNA element and TIA1 RRM2_3. From the bound conformation of SL3, the positions of RRM2 and RRM3 were determined manually according to the found templates in step 1, respectively. The coordinates for RRM2 were from the bound state^24^ (PDB id: 5ith), while those for RRM3 (plus the N-terminal helix α0 in Fig. 1a) were extract from the apo state of TIA1 RRM2_3^23^ (PDB id: 2mjn). Then, the flexible linker between RRM2 and RRM3 was recovered by Modeller^75^. As a result, two possible 3D binding conformations (Conf1 and Conf2) were constructed for subsequent refinement in MD simulations. The final step is to further optimize the binding complex through MD simulations. After balance of the simulation box and release of the position restraints on C1’ (RNA)/Cα (protein) atoms, an extra 100 ns MD simulation with distance restraints (*k*_*b*_ = 800 kJ/mol/nm^2^) on 14 particular atom pairs between RNA and TIA1 was performed. Those atom pair restraints account for key interactions between nucleotide/residue derived from the aforementioned templates in step 1, such as U67@C4-Tyr206@Cδ1, A68@C8-Phe242@Cζ, U76@O2-Arg164@Nη2, U77@O4-Asn169@Nδ2, U78@C4-Phe140@Cδ1, etc. Then, a 2500 ns MD production without any restraints was run to further relax the initial binding complex. For each possible 3D binding conformation in step 3, three duplicated simulations were performed. For each of those six runs (2 conformations × 3 runs), snapshots extracted from the last 1500 ns MD trajectories (7500 snapshots recorded in the duration of 200 ps) were clustered based on gromos method^72^ with a 0.3 nm RMSD cutoff to obtain the most probable conformation and the centroid structure of the largest cluster was chosen. After comprehensive consideration of the profiles of RMSDs (see Supplementary Fig. 7) and occupancies of key interactions between SL3 and TIA1 (see Supplementary Table 6) during simulations, three possible 3D models (see Supplementary Fig. 8 and Supplementary Data 1) were selected as the putative binding complexes for SL3 RNA element and TIA1 RRM2_3 (model #1, #2, and #3 represents the most probable conformation from simulation “Conf1_run1”, “Conf2_run2”, and “Conf1_run2” in Supplementary Fig. 7, respectively). To further validate the stability of the putative 3D binding model (model #1), three additional 1000 ns MD simulations were conducted, and the detailed results were provided in Supplementary Fig. 9 and Supplementary Table 3. In total, more than 18.06 μs collective MD simulations were conducted to extract the putative 3D binding model for SL3 & TIA1 complex. In this procedure, the relative orientation of TIA1 RRM2 and RRM3 was determined by the selected templates (step 1), the predicted bound conformations of SL3 (step 2), and the structure refinement in MD simulations (step 4) together.

### Free energy calculations

The binding affinity changes, due to point mutations of key RNA nts at the interface, between the SL3 RNA element and TIA1 RRM2_3 complex were calculated by the FEP method^40–42^. We estimated the free energy changes for single nucleotide mutation in both the bound state (SL3 and TIA1 complex) $\Delta {G}^{{\rm bound}}$ and the free state (isolated SL3 RNA) $\Delta {G}^{{\rm free}}$ using Gromacs 2020.6. Thus, the binding free energy change caused by nucleotide mutation is estimated as $\Delta \Delta {G}_{{\rm calc}}=\Delta {G}^{{\rm bound}}-\Delta {G}^{{\rm free}}$. For each single nucleotide mutation, the dual-topology file was prepared in a pmx-like^76^ manner based on the Amberff14SB force field^57,58^ (Supplementary Data 2) and eighteen λ windows (0.0, 0.01, 0.05, 0.1, 0.15, 0.2, 0.25, 0.35, 0.45, 0.55, 0.65, 0.75, 0.8, 0.85, 0.9, 0.95, 0.99, and 1.0) with 1.5 ns/window were used. The vdW and electrostatic interactions were transferred simultaneously during simulations and the soft-core potentials (*α* = 0.3) were used. For each mutation, at least five independent runs starting from different conditions were performed for sufficient sampling and at least 270 ns (1.5 ns × 18 windows × 5 runs × 2 states) simulation time was generated, which result in reasonable convergence in the free energy calculations. After MD simulations completion, the Gromacs *bar*^77^ analysis tool was used to estimate the free energy changes based on the last 1 ns simulation per window.

### Vector construction

For the 6 × His-SUMO-TIA1 RRM1-3 (1-274aa) construct, the CDS of TIA1 RRM1-3 was amplified from a human cDNA template using specific primers (Supplementary Table 7) and digested with *Bam*H I/*Hind* III (Thermo Fisher), then ligated into a BamH I/*Hind* III-digested Pet28a-6×His-SUMO vector^76^ to obtain the Pet28a-6 × His-SUMO-Htia1 RRM1-3 construct.

For the 6 × His-SUMO-TIA1 RRM2_3 (93-274aa) construct, the fragment of TIA1 RRM2_3 was amplified from the Pet28a-6 × His-SUMO-TIA1 RRM1-3 plasmid and digested with *Bam*H I/*Hind* III. The resultant fragment was ligated into the *Bam*H I / *Hind* III-digested Pet28a-6 × His-SUMO vector to produce the Pet28a-6×His-SUMO-TIA1 RRM2_3 construct.

### Expression and purification of recombinant proteins

pET28a-6×His-SUMO-TIA1 RRM1-3 and pET28a-6 × His-SUMO-TIA1 RRM2_3 constructs were transformed into *Escherichia coli* strain BL21 (DE3) cells. Cells were grown in Luria–Bertani (LB) at 37 °C until an OD_600nm_ = 0.6–0.8 was reached. Expression of recombinant proteins was typically induced with 0.5 mM IPTG and grown at 16 °C overnight.

For purification of TIA1 RRM2_3, the induced bacterial cells were harvested by centrifugation and re-suspended in ice-cold lysis buffer (50 mM sodium phosphate buffer pH 8.0, 300 mM NaCl, 1 mM PMSF) and disrupted with a high-pressure homogenizer (JNBIO). After centrifugation and filtering with a 0.4 µm filter, the cleared lysate was supplemented with 20 mM imidazole and loaded on a HisTrap HP column (GE Healthcare, Cat#: 17-5248-02). The column was washed with 25 mL wash buffer (Sodium phosphate buffer pH 8.0, 300 mM NaCl, 1 mM PMSF, 80 mM imidazole) and eluted with gradient elution buffer from 80 to 200 mM imidazole (sodium phosphate buffer pH 8.0, 300 mM NaCl, 1 mM PMSF). The peak fractions containing the recombinant 6 × His-SUMO-Htia1 RRM2_3 proteins were pooled and incubated with SUMO protease at 4 °C overnight for the 6 × His-SUMO tag removal. Then the fractions were concentrated by 10 kDa molecular weight cut-off centricon (Thermo Fisher), and loaded onto a HiLoad 26/600 Superdex 200 pg column (GE Healthcare) to separate 6 × His-SUMO tags from the TIA1 RRM2_3 proteins. The gel filtration buffer contains 10 mM Sodium phosphate buffer pH 6.5, 50 mM NaCl, and 2 mM DTT. The peak fractions containing TIA1 RRM2_3 were dialyzed overnight with the dialysis buffer (10 mM sodium phosphate buffer pH 6.5, 50 mM NaCl, 2 mM DTT, 50% glycerol). The purity of the purified TIA1 RRM2_3 proteins was detected on a sodium dodecyl sulfate (SDS) polyacrylamide gel. The TIA1 RRM2_3 proteins were finally frozen by liquid nitrogen and stored at −80 °C. Purification of TIA1 RRM1-3 used the same protocol.

### Electrophoretic mobility shift assays

The EMSA was performed based on previous protocol with minor modifications^78^. Synthesized 5′ end Cy3-labeled RNAs (Supplementary Table 7) were annealed with the annealing buffer (10 mM Tris-HCl pH 7.5, 100 mM KCl) under a predefined procedure: 68 °C for 5 min, then annealing at −0.1 °C/s to 25 °C, and finally at 25 °C for 5 min. Recombinant proteins and annealed Cy3-labeled RNAs were mixed in the EMSA buffer (10 mM Sodium phosphate buffer pH 6.5, 50 mM NaCl, 1 mM DTT, 1 U/μl SUPERase-In Rnase Inhibitor [Thermo Fisher]). Mixtures were incubated at room temperature for 20 min. Bound complexes were added 6× loading buffer (15% Ficoll 400, 0.25% Bromophenol Blue, 0.25% Xylene cyanol, 1× TBE), then resolved on a native 1.2% agarose gel and visualized with iBright1500 (Thermo Fisher). The images were quantified with Image J software. The dissociation constant *K*_d_ for TIA1 RRM2_3 with RNAs were calculated using Prism 8 (GraphPad) software.

### ASO and treatment efficiency

All ASOs were synthesized by Synbio technologies. The sequences of ASOs are listed in Supplementary Table 1. Huh7.5.1 cells, a well differentiated human hepato cellular carcinoma cell line, were plated in 24-well plate at a density of 1.2 × 10^5^ cells per well for 16 h, then 1.5 μl 100 μM ASOs were transfected with 1.5 μl Lipofectamine RNAiMAX (Life technologies, Carlsbad, CA) on a final concentration of 0.3 μM for 12 h. Cells were washed with opti-MEM and incubated with SARS-CoV-2 at an MOI = 0.05 for 1 h, then cells were washed with opti-MEM and supplemented with maintenance medium. At 24 hpi, supernatants were collected and viral RNA in the cell supernatants were extracted by using Direct-zol RNA MiniPrep kit (Zymo Research, CA, USA) according to the manufacturer’s instructions. SARS-CoV-2 viral RNA levels were measured by qPCR.

### Reporting summary

Further information on research design is available in the Nature Portfolio Reporting Summary linked to this article.

## Supplementary information

Supplementary Information

Description of Additional Supplementary Files

Supplementary Data 1

Supplementary Data 2

Reporting Summary

## Data Availability

The data supporting the findings of this study are available from the corresponding authors upon reasonable request. The raw data of EMSA experiments, ASO assessments, and FEP calculations are provided in a Source Data file. The 3D models (in pdb format) constructed in this study are provided in Supplementary Data 1. Other data are included in the main text and the supplemental data. The PDB database used in the study includes PDB IDs: 2l6i, 5ith, 1urn, and 2mjn. Source data are provided with this paper.

## Source data

Source Data

## Code availability

The in-house program used to generate the single-topology files (.top and. gro) for FEP calculations in Gromacs 2020.6 is provided in Supplementary Data 2.
