# Supplementary material for: Mutagenesis and structural studies reveal the basis for the specific binding of SARS-CoV-2 SL3 RNA element with human TIA1 protein
Source: Nat Commun. 2023 Jun 22;14:3715. doi: 10.1038/s41467-023-39410-8 (PMC10287707; doi:10.1038/s41467-023-39410-8)
Supplement: Supplementary file 1 — Supplementary Information [file 41467_2023_39410_MOESM1_ESM.pdf]

Supplementary Information

Mutagenesis and structural studies reveal the basis for the specific binding of SARS-CoV-2 SL3 RNA element with human TIA1 protein

Dong Zhang[1,†], Lulu Qiao[2,†], Xiaobo Lei [3,†], Xiaojing Dong[3], Yunguang Tong[4,5], Jianwei Wang [3,*], Zhiye Wang[2,6,*], and Ruhong Zhou[1,6,*]

[1] Institute of Quantitative Biology, College of Life Sciences, Zhejiang University, Hangzhou, Zhejiang 310058, China

[2] State Key Laboratory of Plant Physiology and Biochemistry, College of Life Sciences, Zhejiang University, Hangzhou, Zhejiang 310058, China

[3] NHC Key Laboratory of Systems Biology of Pathogens and Christophe Mérieux Laboratory, Institute of Pathogen Biology, Chinese Academy of Medical Sciences & Peking Union Medical College, Beijing 100730, China

[4] College of Life Sciences, China Jiliang University, Hangzhou, Zhejiang 310018, China

[5] Department of Pharmacy, China Jiliang University, Hangzhou, Zhejiang 310018, China

[6] The First Affiliated Hospital, College of Medicine, Zhejiang University, Hangzhou, Zhejiang 310058, China

[†] These authors contributed equally: Dong Zhang, Lulu Qiao, and Xiaobo Lei

* To whom correspondence should be addressed: rhzhou@zju.edu.cn (R.Z.); wangzhiye1@zju.edu.cn (Z.W.); wangjw28@163.com (J.W.)

Supplementary Table 1 Sequences of designed ASOs.

ASO Name	Target sequence	Sequence of ASO	Modification sequence[a]
Scramble	--	5'-AATTCTCAAATACGA-3'	/52MOErA/*/i2MOErA/*/i2MOErT/*/i2MOErT//i2MOErC//i2MOErT//i2MOErC//i2MOErA//i2MOErA//i2MOErA//i2MOErT//i2MOErA/*/i2MOErC/*/i2MOErG/*/32MOErA/
ASO-SL2/3	5'-UUUAAAAUCUGUGUGGCUGUC-3'	5'-GACAGCCACACAGATTTTAAA-3'	/52MOErG/*/i2MOErA/*/i2MOErC/*/i2MOErA//i2MOErG//i2MOErC//i2MOErC//i2MOErA//i2MOErC//i2MOErA//i2MOErC//i2MOErA//i2MOErG//i2MOErA//i2MOErT//i2MOErT//i2MOErT//i2MOErT/*/i2MOErA/*/i2MOErA/*/32MOErA/
ASO-HP	5'-AGAUCUGUUCUCUAA-3'	5'-TTAGAGAACAGATCT-3'	/52MOErT/*/i2MOErT/*/i2MOErA/*/i2MOErG//i2MOErA//i2MOErG//i2MOErA//i2MOErA//i2MOErC//i2MOErA//i2MOErG//i2MOErA/*/i2MOErT/*/i2MOErC/*/32MOErT/
ASO-TL	5'-UUUAAAAUCUGUGUGG-3'	5'-CCACACAGATTTTAAA-3'	/52MOErC/*/i2MOErC/*/i2MOErA/*/i2MOErC//i2MOErA//i2MOErC//i2MOErA//i2MOErG//i2MOErA//i2MOErT//i2MOErT//i2MOErT//i2MOErT/*/i2MOErA/*/i2MOErA/*/32MOErA/
ASO-TRS	5'-UCUAAACGAACUUUA-3'	5'-TAAAGTTCGTTTAGA-3'	/52MOErT/*/i2MOErA/*/i2MOErA/*/i2MOErA//i2MOErG//i2MOErT//i2MOErT//i2MOErC//i2MOErG//i2MOErT//i2MOErT//i2MOErT/*/i2MOErA/*/i2MOErG/*/32MOErA/

[a]2MOE is the 2'-O-methoxyethyl (2'-MOE) and * is the phosphorothioate backbone modification.

Supplementary Table 2 Percent yield of the bona fide SARS-CoV-2 RNA virus and cell viability transfected with designed ASOs in Huh7.5.1 cells for 24 h relative to the "scramble" control treated with a non-targeting ASO.

Replicate	scramble	ASO-SL2/3	ASO-HP	ASO-TL	ASO-TRS
**Percent yield of the bona fide SARS-CoV-2 RNA virus**					
1	103.4	51.5	48.2	53.6	7.3
2	100.9	57.9	44.8	58.1	7.3
3	95.2	47.7	41.5	43.5	18.1
4	98.3	--	--	--	--
5	102.1	--	--	--	--
mean±s.d.	100±3.3	52.4±5.2	44.9±3.3	51.8±7.5	10.9±6.2
**Cell viability**					
1	101.1	115.5	115.0	103.4	116.5
2	87.0	118.6	96.8	98.7	116.3
3	123.3	116.5	109.8	94.6	110.6
mean±s.d.	103.8±18.3	116.8±1.6	107.2±9.4	98.9±4.4	114.5±3.3

Supplementary Table 3 Comparison of occupancies (%) of key interactions between RNA element and protein in different simulation systems. Only the last 500 ns simulations were considered.

Interaction	SL3&TIA1	SL3&TIAR	SL3_A68U &TIA1	SL3_A69C &TIA1
U67@N3-H3...Trp272@O[a]	69.2	72.0	77.3	75.9
Lys274@N-H...U67@O2	24.8	34.5	31.1	33.2
Asp101@N-H...U76@O2	32.1	31.2	34.4	42.2
U76@N3-H3...Asp101@Oδ1	18.3	21.7	25.2	20.0
Arg164@Nη-Hη...U76@O4	34.7	38.4	35.3	37.7
Arg167@Nη-Hη...U76@O2’	40.8	35.8	38.4	44.9
Asn169@Nδ2-Hδ2...U77@O4	48.4	43.7	46.4	38.5
U77@N3-H3...Trp170@O	71.6	71.3	72.3	77.5
Arg125@Nε-Hε/Nη-Hη...U78@O2	35.8	35.8	46.4	44.3
His96@Nε2-Hε2...U78@O4	34.0	21.8	35.8	32.6
Arg233@Nε-Hε...A68@N3	5.4	28.6	--	1.4
Arg233@Nη-Hη...A69@N3	4.5	6.2	3.8	--
Arg233@Nε-Hε...U68@O2	--	--	22.5	--
Arg233@Nη-Hη...C69@O2	--	--	--	45.4
U67--Tyr206[b]	98.1	92.4	97.0	96.1
U67--Lys274	8.1	16.1	20.4	9.6
A68--Phe242	98.6	89.7	97.9	97.3
U77--Phe98	94.4	95.1	95.8	97.1
U78--Phe140	99.4	100.0	99.9	99.3
Percentage of structures in the largest cluster[c]	98.9	100.0	100.0	99.8

[a]Hydrogen bonds were calculated by VMD with donor-acceptor distance < 3.5 Å and angle < 30°.

[b]Aromatic stacking interactions were determined with distance of center-of-mass between nucleotide base moiety (only heavy-atom) and residue side chain (only heavy-atom and except Cβ atom) < 4.5 Å.

[c]Structure snapshots were clustered by gromos method with pairwise RMSD cutoff of 3 Å.

Supplementary Table 4 The relative binding free energy changes for single nucleotide mutations on SL3 binding. Mean ± standard deviation (kcal/mol) from five independent FEP runs were given.

Mutation	FEP (model #1)			$\Delta\Delta G_{exp}$	FEP (model #2)	
	ΔG^{bound}	ΔG^{free}	$\Delta\Delta G_{calc}$		ΔG^{bound}	$\Delta\Delta G_{calc}$
U67A	-10.92±0.41	-13.72±0.14	2.80±0.44	--	--	--
U67C	-96.86±0.26	-100.48±0.20	3.62±0.33	0.85±0.21	-99.66±0.58	0.82±0.62
U67G	-60.12±0.50	-63.96±0.30	3.84±0.58	--	--	--
A68C	-85.47±0.81	-86.10±0.26	0.64±0.86	-0.04±0.10	-84.72±0.23	1.39±0.35
A68G	-51.90±0.62	-51.50±0.28	-0.40±0.68	0.09±0.10	-50.57±0.26	0.93±0.38
A68U	14.78±0.59	16.62±0.20	-1.84±0.63	-0.31±0.10	15.53±0.63	-1.08±0.66
A69C	-87.14±0.11	-86.70±0.17	-0.45±0.21	-0.37±0.09	-86.14±0.31	0.55±0.36
A69G	-51.93±0.64	-48.85±0.12	-3.07±0.65	--	--	--
A69U	14.64±0.15	18.07±0.16	-3.43±0.22	--	--	--
U76A	-12.74±0.42	-15.86±0.85	3.11±0.95	1.22±0.20	-10.10±0.12	5.76±0.85
U76C	-98.33±0.21	-98.10±0.52	-0.23±0.56	--	--	--
U76G	-61.03±0.52	-65.60±0.48	4.56±0.71	0.94±0.21	-60.10±0.41	5.49±0.63
U77A	-10.75±0.37	-13.17±0.70	2.42±0.80	1.05±0.22	-14.65±0.38	-1.49±0.80
U77C	-96.89±0.39	-99.18±0.81	2.29±0.91	0.29±0.11	-98.76±0.86	0.42±1.19
U77G	-60.30±0.39	-63.59±0.44	3.29±0.59	1.18±0.17	-63.41±0.69	0.18±0.82
U78A	-10.09±0.57	-13.71±0.21	3.62±0.61	1.34±0.19	-11.35±0.59	2.36±0.63
U78C	-98.43±0.27	-100.61±0.61	2.18±0.66	0.32±0.11	-98.96±0.28	1.65±0.67
U78G	-59.79±0.38	-64.01±0.34	4.22±0.51	--	--	--

Supplementary Table 5 Summary of simulation systems in this study.

Name	Number of atoms	Simulation time	Description
SL2 (3D)	95	50 ns×10 replicas replica-exchange MD, 2 runs	Predict RNA 3D structure in the coarse-grained IsRNA2 model for SL2 RNA element
SL3 (3D)	105	50 ns×10 replicas replica-exchange MD	Predict RNA 3D structure in the coarse-grained IsRNA2 model for SL3 RNA element
SL3 (free)	19,642	1,500 ns, 3 runs	Relax the 3D structure of SL3 in all-atom MD simulations to prepare FEP free state
SL3 & TIA1 (Conf1)	57,936	2,500 ns, 3 runs	Relax the 3D binding complex of SL3 & TIA1 from initial conformation #1
SL3 & TIA1 (Conf2)	61,973	2,500 ns, 3 runs	Relax the 3D binding complex of SL3 & TIA1 from initial conformation #2
SL3 & TIA1 (stability)	57,936	1,000 ns, 3 runs	Validate the stability of the putative SL3 & TIA1 3D binding model (model #1)
SL3_allC & TIA1	57,932	1,500 ns, 3 runs	Simulate the binding complex of SL3 all C's variant & TIA1
SL3 & TIAR	57,947	1,500 ns, 3 runs	Simulate the binding complex of SL3 and related TIAR
SL3_A68U & TIA1	57,933	1,000 ns, 3 runs	Simulate the binding complex of SL3 A68U mutation & TIA1
SL3_A69C & TIA1	57,934	1,000 ns, 3 runs	Simulate the binding complex of SL3 A69C mutation & TIA1

Supplementary Table 6 Occupancies (%) of key interactions between SL3 RNA element and TIA1 protein in the last 1500 ns MD simulations (to extract the putative 3D structure for binding complex of SL3 & TIA1).

Interaction	Conf1			Conf2		
	run1	run2	run3	run1	run2	run3
U67@N3-H3...Trp272@O[a]	74.0	81.5	72.8	54.9	73.9	80.1
Lys274@N-H...U67@O2	28.8	26.5	36.2	26.7	33.6	40.0
Asp101@N-H...U76@O2	29.6	68.2	0.0	53.4	48.8	35.2
U76@N3-H3...Asp101@Oδ1	25.7	22.5	0.0	32.3	56.3	67.6
Arg164@Nη-Hη...U76@O4	41.5	40.7	0.0	10.2	33.3	48.6
Arg167@Nη-Hη...U76@O2’	35.7	66.3	0.0	40.2	30.8	30.0
Asn169@Nδ2-Hδ2...U77@O4	47.4	52.7	45.2	60.0	56.6	28.8
U77@N3-H3...Trp170@O	63.6	68.3	47.5	71.8	27.0	61.2
Arg125@Nε-Hε/Nη-Hη...U78@O2	46.6	44.9	63.3	39.4	13.0	4.1
His96@Nε2-Hε2...U78@O4	36.7	30.7	0.0	0.2	0.6	0.8
U67--Tyr206[b]	95.8	85.7	96.0	97.4	97.5	90.1
U67--Lys274	12.6	25.6	19.9	11.2	11.5	14.8
A68--Phe242	93.3	57.2	79.3	64.3	99.3	76.4
U77--Phe98	96.6	94.5	93.5	92.8	96.6	86.2
U78--Phe140	99.6	84.6	100.0	99.0	42.2	61.6
Percentage of structures in the largest cluster[c]	99.4	99.5	98.7	46.0	96.2	73.8

[a]Hydrogen bonds were calculated by VMD with donor-acceptor distance < 3.5 Å and angle < 30°.

[b]Aromatic stacking interactions were determined with distance of center-of-mass between nucleotide base moiety (only heavy-atom) and residue side chain (only heavy-atom and except Cβ atom) < 4.5 Å.

[c]Structure snapshots were clustered by gromos method with pairwise RMSD cutoff of 3 Å.

Supplementary Table 7 Sequences of primers.

Primers for pET28a-His-SUMO-TIA1 (93-274) and TIA1 (93-274)	
	5'-3'
hTIA1-For 1 BamH1	aaaggatccGAGGACGAGATGCCCAAGACTCT
hTIA1-For 93 BamH1	aaaggatccAATCATTTCCATGTCTTTGTTGG
hTIA1-Rev 274 Hind3	gtgaagcttcaTTTGCCCCAATAGCATTTCACAAC
Cy3 labelled RNA probes	
U11	Cy3-UUUUUUUUUUU
C21	Cy3-CCCCCCCCCCCCCCCCCCCCC
SL2+SL3	Cy3-UUUCGAUCUCUUGUAGAUCUGUUCUCUAAACGAACUUUAAAAUCU
SL2	Cy3-UUUCGAUCUCUUGUAGAUC
SL3	Cy3-UGUUCUCUAAACGAACUUUAA
SL3-HP3	Cy3-UGUUCUAAGAACUUUAA
SL3-HP-C7	Cy3-UGUUCCCCCCCGAACUUUAA
SL3-HP-U7	Cy3-UGUUCUUUUUUUGAACUUUAA
SL3-TL1	Cy3-UGUUCUCUAAACGAACU
SL3-TL-C3	Cy3-UGUUCUCUAAACGAACCCCAA
SL3-TL-G3	Cy3-UGUUCUCUAAACGAACGGGAA
SL3-GC	Cy3-UGGGCUCUAAACGCCCUUUAA
U67C	Cy3-UGUUCUCCAAACGAACUUUAA
A68C	Cy3-UGUUCUCUCAACGAACUUUAA
A68G	Cy3-UGUUCUCUGAACGAACUUUAA
A68U	Cy3-UGUUCUCUUAACGAACUUUAA
A69C	Cy3-UGUUCUCUACACGAACUUUAA
U76A	Cy3-UGUUCUCUAAACGAACAUUAA
U76G	Cy3-UGUUCUCUAAACGAACGUUAA
U77A	Cy3-UGUUCUCUAAACGAACUAUAA
U77C	Cy3-UGUUCUCUAAACGAACUCUAA
U77G	Cy3-UGUUCUCUAAACGAACUGUAA
U78A	Cy3-UGUUCUCUAAACGAACUUAAA
U78C	Cy3-UGUUCUCUAAACGAACUUCAA
RousettusHKU9 SL3	Cy3-UCACUCUUGAACGAACUUAAA
MERS SL3	Cy3-UUUGAUUUUAACGAACUUAAA

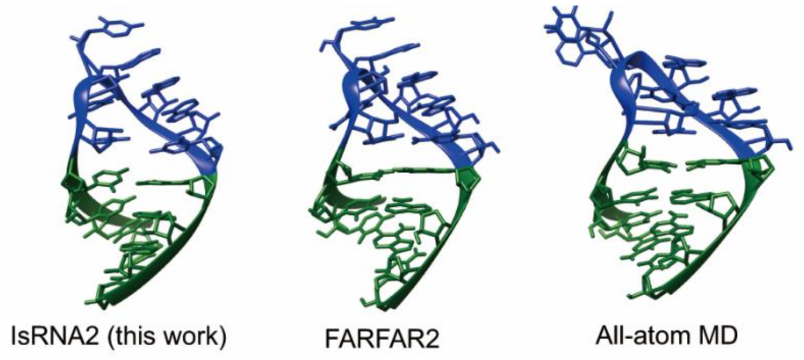

Supplementary Fig. 1 Comparison of the top 3D structures predicted by IsRNA2, FARFAR2, and all-atom MD simulations for SL3 RNA element. The heavy-atom RMSDs are 1.4 (IsRNA2 vs. FARFAR2) and 2.4 Å (IsRNA2 vs. All-atom MD).

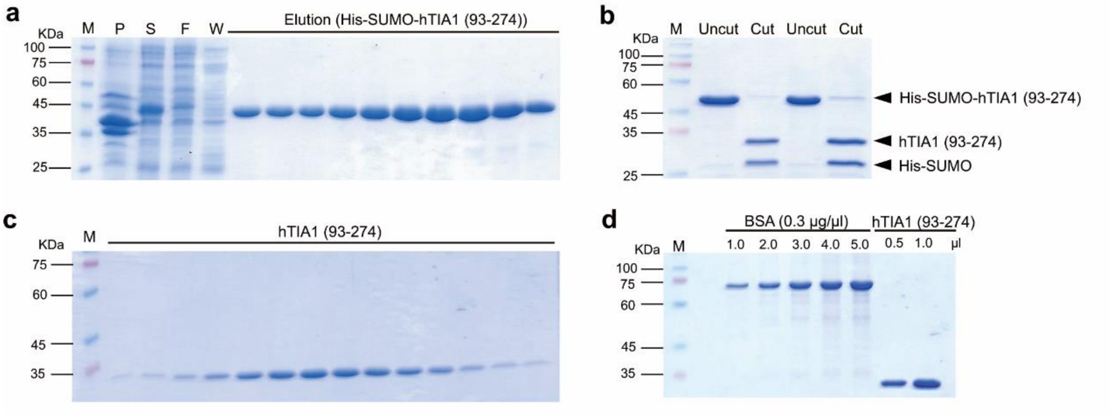

Supplementary Fig. 2 High purity of recombinant TIA1 proteins. **a** His-SUMO-TIA1 (93-274aa) was purified by Ni-NTA affinity chromatography. **b** His-SUMO tag was cut by SUMO proteinase. **c** TIA1 (93-274aa) was further purified through size exclusive chromatography. **d** Gel image of recombinant TIA1 (93-274aa) proteins with two-step purification. Source data are provided as a Source Data file.

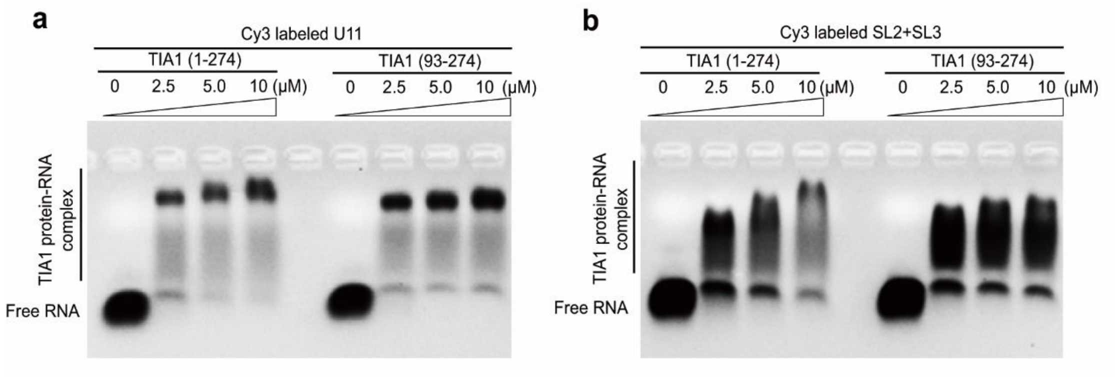

Supplementary Fig. 3 EMSA shows full TIA1 RRM1-3 (1-274aa) and truncated TIA1 RRM2_3 (93-274aa) have comparable binding affinity with 5'-end Cy3-labelled (**a**) OligoU (U11) and (**b**) SL2+SL3 RNA. Source data are provided as a Source Data file.

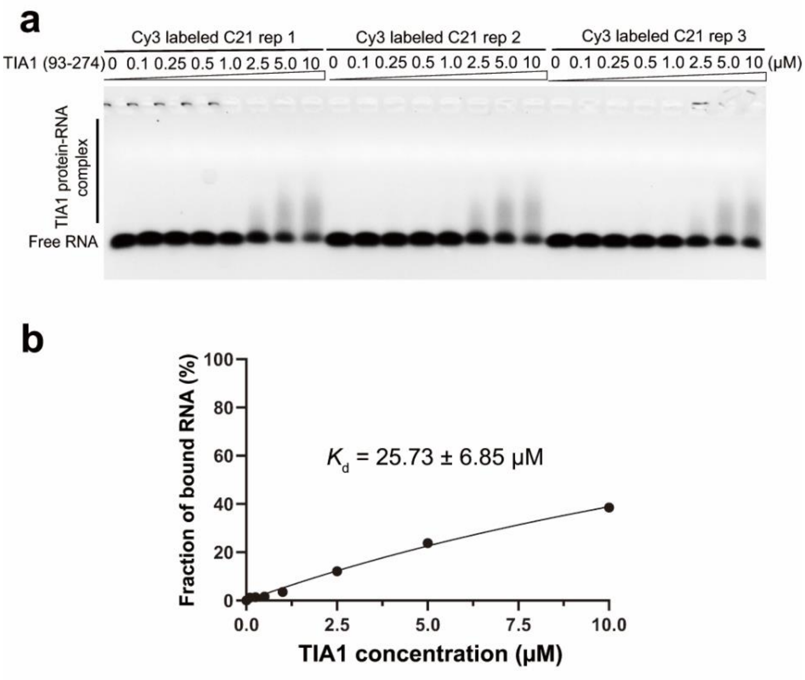

Supplementary Fig. 4 A faint binding of TIA1 to 5'-end Cy3-labelled OligoC (C21) RNA: (**a**) EMSA results (three replicates) and (**b**) the binding curve. The K_d values were calculated from the EMSA image quantification from three independent experiments. Data are presented as mean ± SD. Source data are provided as a Source Data file.

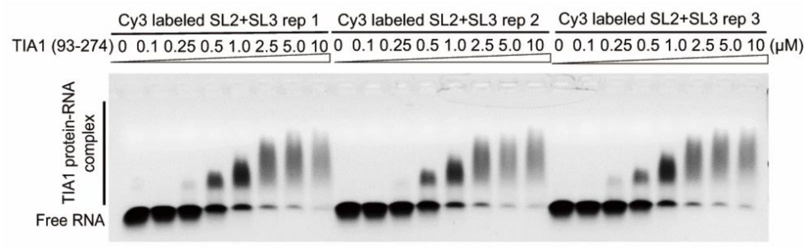

Supplementary Fig. 5 EMSA results (three replicates) show the mobility pattern of the TIA1 & SL2+SL3 RNA complex. The gel image of the first replicate (rep 1) is also shown in Fig. 1d.

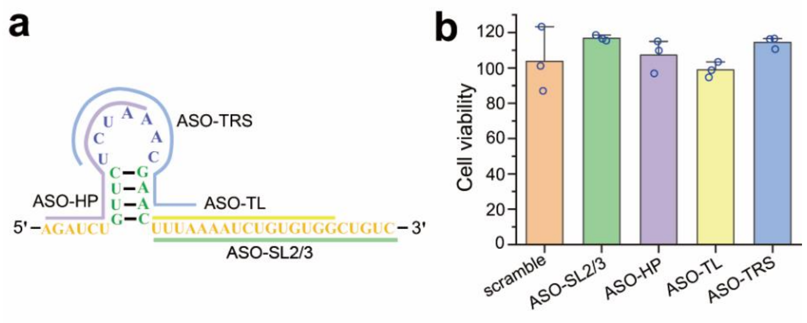

Supplementary Fig. 6 Designed ASOs and their cell cytotoxicity. **a** Schematic of ASOs design. **b** Cell viability of Huh7.5.1 cells transfected with designed ASOs. Data represent the mean ± SEM; n = 3 biological replicates.

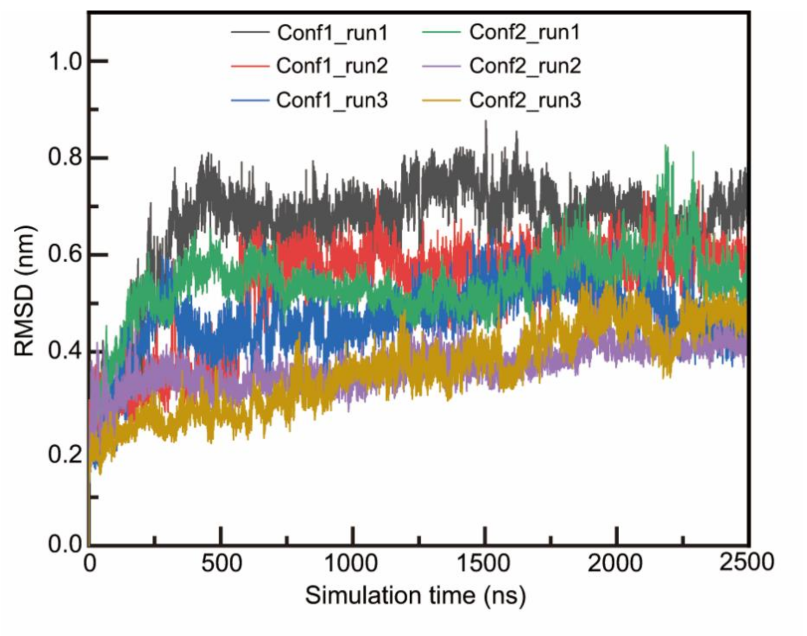

Supplementary Fig. 7 Heavy-atom RMSDs as functions of simulation time for two constructed binding conformations (Conf1 and Conf2) of SL3 RNA element and TIA1 protein. Three 2500-ns independent runs (run1, run2, and run3) were performed for each construction.

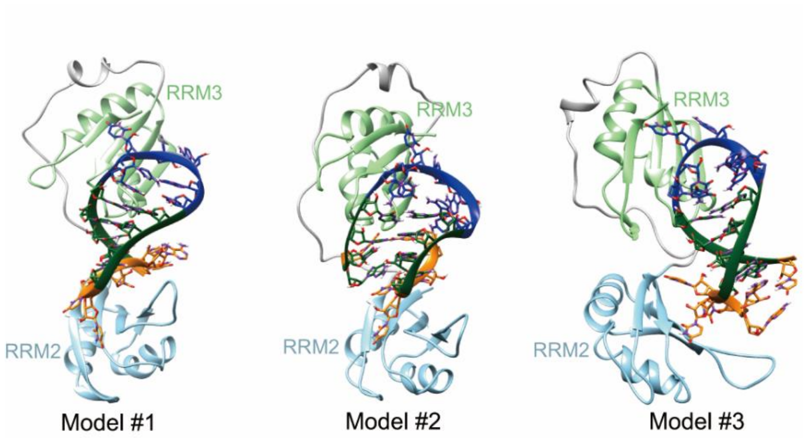

Supplementary Fig. 8 Comparison of the three top 3D models (#1-3) for binding complex of SL3 RNA element with TIA1 protein constructed by computational modeling (#1 is the same as the most probable model in Fig. 3a in the main text). TIA1 RRM2, RRM3, and flexible linker are colored by sky blue, light green, and gray, respectively. Hairpin loop, stem, and terminal loop of SL3 are separately colored by blue, green, and orange.

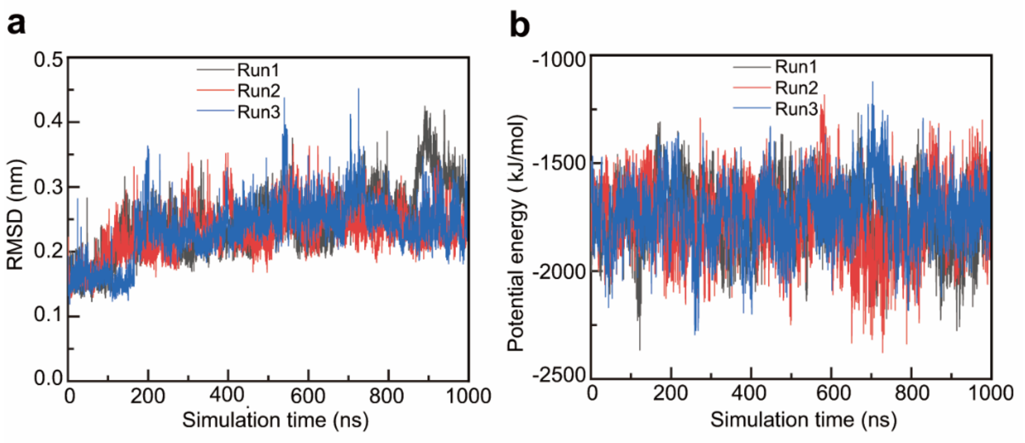

Supplementary Fig. 9 Validation the stability of the putative 3D binding model (model #1) for SL3 and TIA1 complex: (**a**) Heavy-atom RMSDs and (**b**) potential energies between SL3 RNA and TIA1 protein as functions of simulation time for extended MD simulations. Three 1000-ns independent simulations were run.

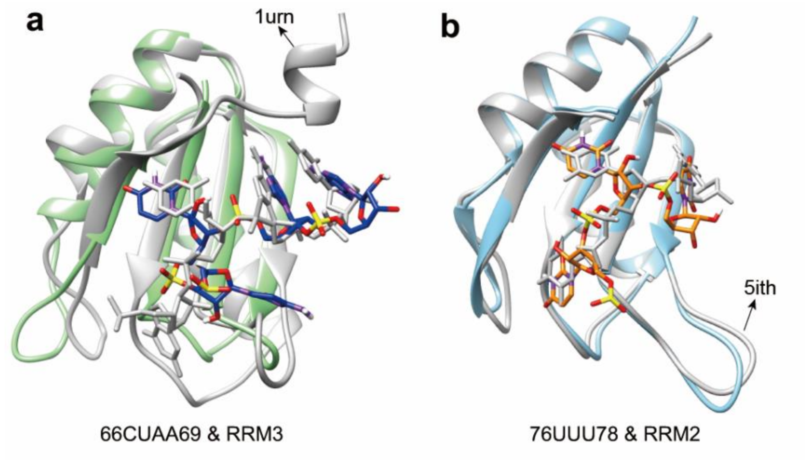

Supplementary Fig. 10 Comparison of the putative 3D model of SL3 and TIA1 RRM23 binding complex (model #1) with selected templates. **a** 66CUAA69 RNA segment of SL3 hairpin loop & TIA1 RRM3 vs. an RNA hairpin complexed with RRM of U1A spliceosomal protein (light gray, PDB id: 1urn). **b** 76UUU78 RNA segment of SL3 3'-terminal loop & TIA1 RRM2 vs. 5'-TTT-3' oligonucleotide binding to TIA1 RRM2 (light gray, PDB id: 5ith). Atoms of phosphorus, oxygen, nitrogen, and hydrogen are colored yellow, red, purple, and white, respectively, and carbon atoms are colored according to their locations.

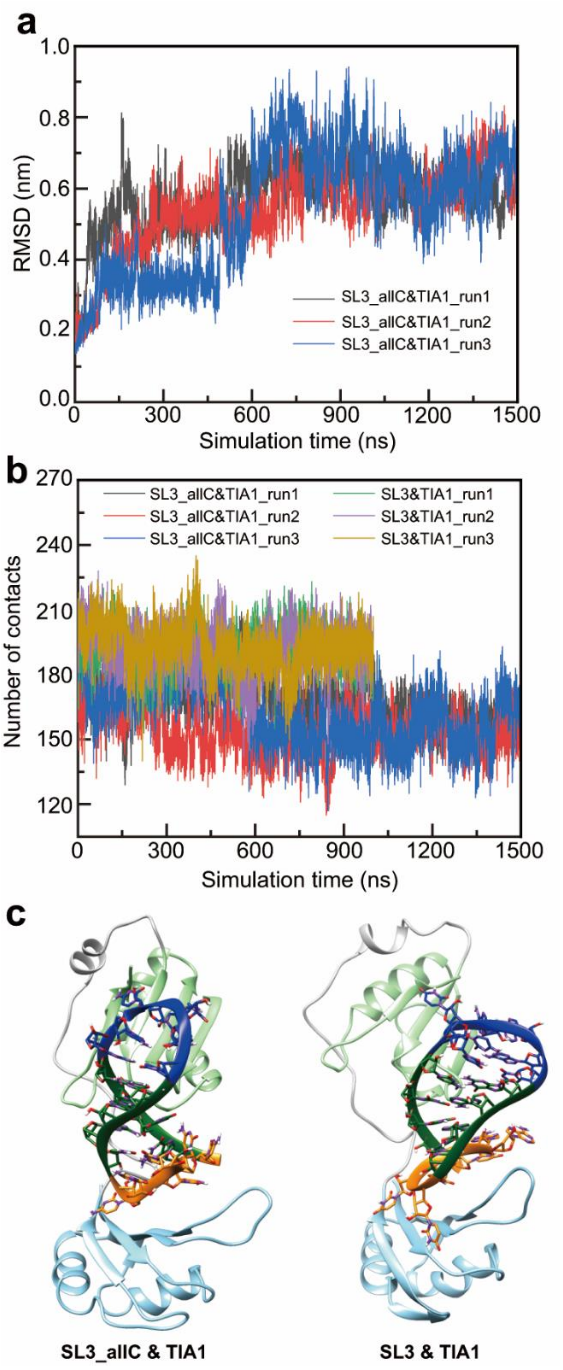

Supplementary Fig. 11 Characterization of binding properties between SL3 all C's variant (SL3_allC) and TIA1. **a** Heavy-atom RMSDs as functions of simulation time for binding model of SL3_allC and TIA1. This all C's variant replaces all nucleotides in the loop region of SL3 by cytosines (5'-CGUUCCCCCCCCGAACCCCCC-3') and is thought to have a much weaker binding ability to TIA1 protein than wild-type SL3 RNA element. Three independent runs were performed. **b** Comparison of the numbers of contacts (< 5 Å) between RNA and protein in the course of simulations for SL3_allC variant & TIA1 and wild type SL3 & TIA1. Number of contacts for SL3_allC & TIA1 (~156) is apparently less than that for SL3 & TIA1 (~190). **c** Comparison of 3D binding models extracted from MD simulations for SL3_allC & TIA1 and SL3 & TIA1 (model #1). The colors are same as those in Fig. 3.

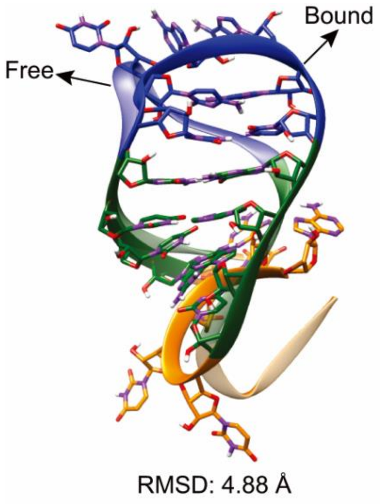

Supplementary Fig. 12 Comparison of 3D structures of SL3 RNA element in the bound (complexed with TIA1) and free states (in transparent). The heavy-atom RMSD between two states is 4.9 Å. Hairpin loop, stem, and terminal loop of SL3 are colored by blue, green, and orange, respectively. Atoms of oxygen, nitrogen, and hydrogen are colored by red, purple, and white, respectively, and carbon atoms are colored according to their locations.

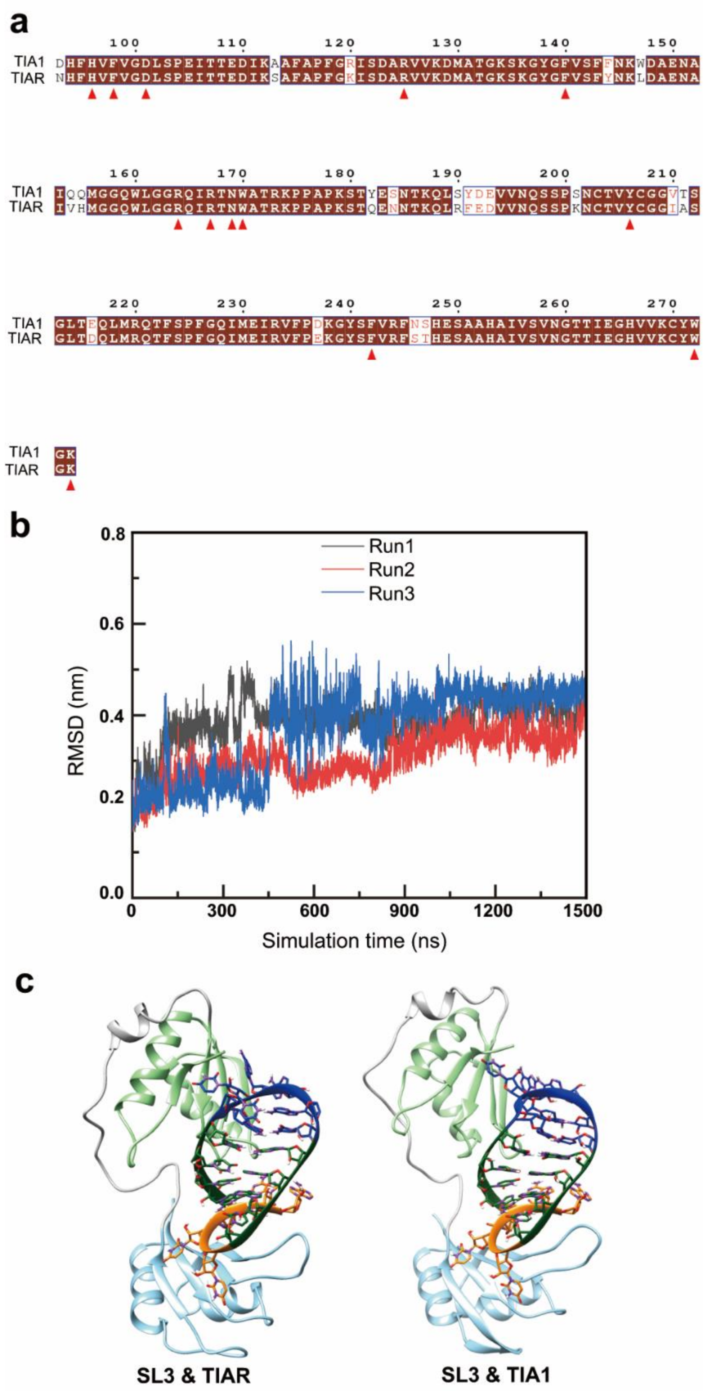

Supplementary Fig. 13 Characterization of binding properties between TIA1 related protein TIAR and SL3. **a** Sequence alignment of TIA1 (93-274 aa) and its related protein TIAR (95-276 aa). Key residues that interact with SL3 RNA element are marked by red triangles. **b** Heavy-atom RMSDs as functions of simulation time for SL3 & TIAR. Three independent runs were performed. **c** Comparison of 3D binding models extracted from MD simulations for SL3 & TIAR and SL3 & TIA1 (model #1). The colors are same as those in Fig. 3.

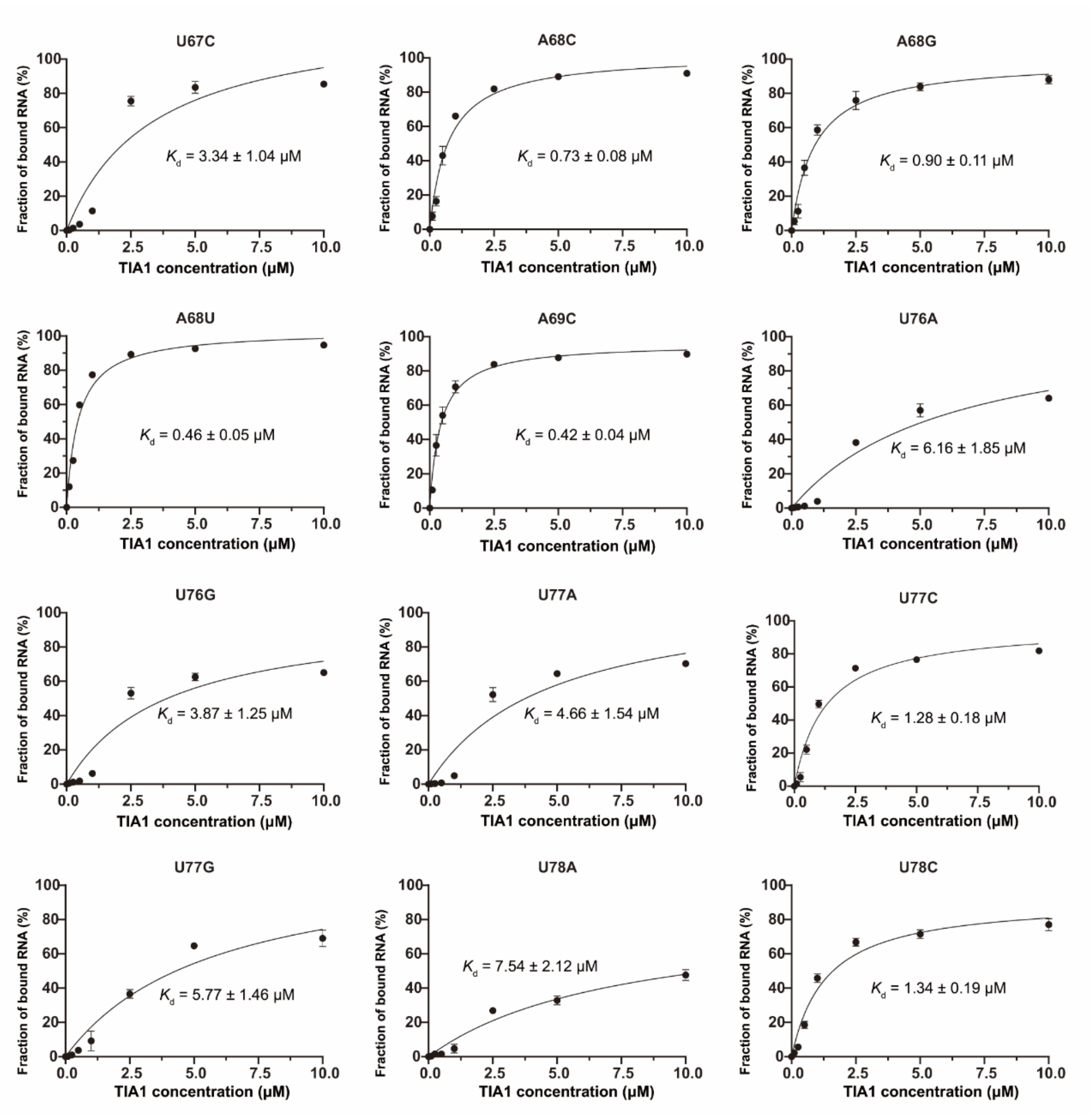

Supplementary Fig. 14 The binding curves of TIA1 to single-nucleotide mutated variants of SL3 RNAs. The K_d values were calculated from the EMSA image quantification from three independent experiments. Data are presented as mean ± SD. Source data are provided as a Source Data file.

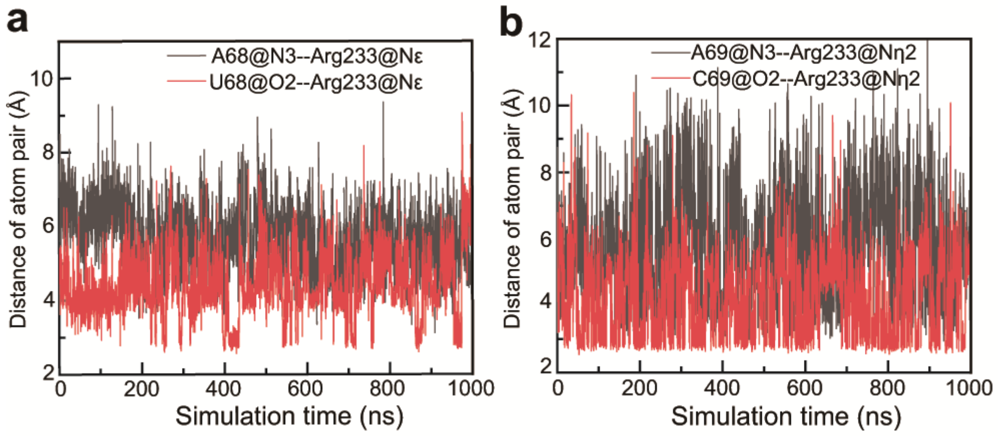

Supplementary Fig. 15 Distances of listed atom pairs as functions of simulation time for SL3 (**a**) A68U and (**b**) A69C mutations. Decreased pair distances enable the formation of an extra hydrogen bond between side chain of Arg233 in TIA1 RRM3 and nucleotide U68/C69 in SL3 hairpin loop.
