# Supplementary material for: Mutagenesis and structural studies reveal the basis for the specific binding of SARS-CoV-2 SL3 RNA element with human TIA1 protein
Source: Nat Commun. 2023 Jun 22;14:3715. doi: 10.1038/s41467-023-39410-8 (PMC10287707; doi:10.1038/s41467-023-39410-8)
Supplement: Supplementary file 2 — Description of Additional Supplementary Files [file 41467_2023_39410_MOESM2_ESM.pdf]

Description of Additional Supplementary Files

File Name: Supplementary Data 1
Description: The 3D models (in pdb format) constructed in this study.

File Name: Supplementary Data 2
Description: The in-house program used to generate the singletopology files (.top and .gro) for FEP calculations in Gromacs.
