# Supplementary material for: Mutagenesis and structural studies reveal the basis for the specific binding of SARS-CoV-2 SL3 RNA element with human TIA1 protein
Source: Nat Commun. 2023 Jun 22;14:3715. doi: 10.1038/s41467-023-39410-8 (PMC10287707; doi:10.1038/s41467-023-39410-8)
Supplement: Supplementary file 5 — Reporting Summary [file 41467_2023_39410_MOESM5_ESM.pdf]

nature portfolio

Corresponding author(s): Jianwei Wang , Zhiye Wang, and Ruhong Zhou

Last updated by author(s): May 26, 2023

Reporting Summary

Nature Portfolio wishes to improve the reproducibility of the work that we publish. This form provides structure for consistency and transparency in reporting. For further information on Nature Portfolio policies, see our Editorial Policies and the Editorial Policy Checklist.

Statistics

For all statistical analyses, confirm that the following items are present in the figure legend, table legend, main text, or Methods section.

n/a	Confirmed	
☐	☒	The exact sample size (*n*) for each experimental group/condition, given as a discrete number and unit of measurement
☐	☒	A statement on whether measurements were taken from distinct samples or whether the same sample was measured repeatedly
☐	☒	The statistical test(s) used AND whether they are one- or two-sided<br>*Only common tests should be described solely by name; describe more complex techniques in the Methods section.*
☒	☐	A description of all covariates tested
☒	☐	A description of any assumptions or corrections, such as tests of normality and adjustment for multiple comparisons
☐	☒	A full description of the statistical parameters including central tendency (e.g. means) or other basic estimates (e.g. regression coefficient) AND variation (e.g. standard deviation) or associated estimates of uncertainty (e.g. confidence intervals)
☐	☒	For null hypothesis testing, the test statistic (e.g. *F*, *t*, *r*) with confidence intervals, effect sizes, degrees of freedom and *P* value noted<br>*Give P values as exact values whenever suitable.*
☒	☐	For Bayesian analysis, information on the choice of priors and Markov chain Monte Carlo settings
☒	☐	For hierarchical and complex designs, identification of the appropriate level for tests and full reporting of outcomes
☐	☒	Estimates of effect sizes (e.g. Cohen's *d*, Pearson's *r*), indicating how they were calculated

Our web collection on statistics for biologists contains articles on many of the points above.

Software and code

Policy information about availability of computer code

Data collection	IsRNA2, Gromacs 2020.6, Modeller 10.1, AmberTools22
Data analysis	VMD 1.9.4a51, UCSF Chimera 1.15, Gromacs 2020.6, OriginPro 2021, GraphPad Prism 8.3.0.538, Image J 1.41, iBright Analysis Software 3.1.3

For manuscripts utilizing custom algorithms or software that are central to the research but not yet described in published literature, software must be made available to editors and reviewers. We strongly encourage code deposition in a community repository (e.g. GitHub). See the Nature Portfolio guidelines for submitting code & software for further information.

Data

Policy information about availability of data

All manuscripts must include a data availability statement. This statement should provide the following information, where applicable:

- Accession codes, unique identifiers, or web links for publicly available datasets
- A description of any restrictions on data availability
- For clinical datasets or third party data, please ensure that the statement adheres to our policy

Source data are provided with this paper. The raw data of EMSA experiments, ASO assessments, and FEP calculations are provided in Source Data file. The 3D models (in pdb format) constructed in this study are provided in Supplementary Data 1. Other data are included in the main text and the supplemental data. The PDB database used in the study includes PDB IDs: 2l6i, 5ith, 1urn, and 2mjn.

Research involving human participants, their data, or biological material

Policy information about studies with human participants or human data. See also policy information about sex, gender (identity/presentation), and sexual orientation and race, ethnicity and racism.

Reporting on sex and gender	This information has not been collected
Reporting on race, ethnicity, or other socially relevant groupings	This information has not been collected
Population characteristics	This information has not been collected
Recruitment	This information has not been collected
Ethics oversight	This information has not been collected

Note that full information on the approval of the study protocol must also be provided in the manuscript.

Field-specific reporting

Please select the one below that is the best fit for your research. If you are not sure, read the appropriate sections before making your selection.

☒ Life sciences ☐ Behavioural & social sciences ☐ Ecological, evolutionary & environmental sciences

For a reference copy of the document with all sections, see nature.com/documents/nr-reporting-summary-flat.pdf

Life sciences study design

All studies must disclose on these points even when the disclosure is negative.

Sample size	Standardized sample sizes were used. Three biological replicates were performed for EMSA and ASO assessments.
Data exclusions	No data were excluded
Replication	All experiments were repeated at least three times and reliably reproduced. The reproducibility of the experimental findings was reflected by statistics including the averages with error bars as well as the individual data points in the figures and graphs.
Randomization	Randomization was not generally used in this study. No human or animal subjects were used in this study.
Blinding	Blinding was not necessary in this study since no subjective allocation was involved.

Reporting for specific materials, systems and methods

We require information from authors about some types of materials, experimental systems and methods used in many studies. Here, indicate whether each material, system or method listed is relevant to your study. If you are not sure if a list item applies to your research, read the appropriate section before selecting a response.

Materials & experimental systems

n/a	Involved in the study	
☒	☐	Antibodies
☐	☒	Eukaryotic cell lines
☒	☐	Palaeontology and archaeology
☒	☐	Animals and other organisms
☒	☐	Clinical data
☒	☐	Dual use research of concern
☒	☐	Plants

Methods

n/a	Involved in the study	
☒	☐	ChIP-seq
☒	☐	Flow cytometry
☒	☐	MRI-based neuroimaging

Eukaryotic cell lines

Policy information about cell lines and Sex and Gender in Research

Cell line source(s)	ATCC, human liver cancer cell line Huh7.5.1 cells

Authentication	Cell lines were obtained from ATCC without independent validation.
Mycoplasma contamination	All cell lines were negative for Mycoplasma contamination.
Commonly misidentified lines (See ICLAC register)	No commonly misidentified cell lines were used in this study.
